# Comprehensive analysis of transcription factor binding sites and expression profiling of rice pathogenesis related genes (*OsPR1*)

**DOI:** 10.3389/fpls.2024.1463147

**Published:** 2024-10-25

**Authors:** Diksha Kumari, Bishun Deo Prasad, Padmanabh Dwivedi, Sangita Sahni, Mankesh Kumar, Saud Alamri, Muhammad Faheem Adil, Khaled A. Alakeel

**Affiliations:** ^1^ Department of Plant Physiology, Institute of Agricultural Sciences, Banaras Hindu University, Varanasi, UP, India; ^2^ Department of Agricultural Biotechnology & Molecular Biology, College of Basic Sciences and Humanities (CBS&H), Dr. Rajendra Prasad Central Agricultural University, Pusa, Samastipur, Bihar, India; ^3^ Department of Plant Pathology, Tirhut College of Agriculture (TCA), Dr. Rajendra Prasad Central Agricultural University, Pusa, Samastipur, Bihar, India; ^4^ Department of Plant Breeding & Genetics, Bihar Agricultural University, Sabour, Bhagalpur, Bihar, India; ^5^ Department of Botany and Microbiology, College of Science, King Saud University, Riyadh, Saudi Arabia; ^6^ Zhejiang Key Laboratory of Crop Germplasm Resource, Department of Agronomy, College of Agriculture and Biotechnology, Zhejiang University, Hangzhou, China; ^7^ Department: Advanced Agricultural & Food Technologies Institute, Sustainability and Environment Sector, King Abdulaziz City for Science and Technology, Riyadh, Saudi Arabia

**Keywords:** Pathogenesis-related protein1 (PR1), Brassinosteroids (BR), Heat stress (HS), Promoter, Transcription Factor Binding Sites (TFBSs), Cysteine-rich secretory protein (CAP)

## Abstract

Pathogenesis-related (PR) proteins, found in plants, play a crucial role in responding to both biotic and abiotic stresses and are categorized into 17 distinct families based on their properties and functions. We have conducted a phylogenetic analysis of *OsPR1* genes (rice *PR1* genes) in conjunction with 58 putative *PR1* genes identified in *Brachypodium distachyon*, *Hordeum vulgare*, *Brassica rapa*, and *Zea mays* through BLASTP predictions. We extensively investigated the responses of the remaining 11 rice *PR1* genes, using *OsPR1a* as a reference, under various stress conditions, including phytohormone treatments (salicylic acid and brassinosteroid [BR]), wounding, and heat stress (HS). In rice, of the 32 predicted *OsPR1* genes, 12 have been well-characterized for their roles in disease resistance, while the functions of the remaining genes have not been studied extensively. In our study, we selected an additional 11 *OsPR1* genes for further analysis and constructed a phylogenetic tree based on the presence of a 10-amino-acid-long conserved motif within these proteins. The phylogenetic analysis revealed that both *OsPR1a* from earlier studies and *OsPR1-74* from our current study belong to the same clade. These genes consistently exhibit upregulation in response to diverse stress treatments such as biotic stress and abiotic stresses such as heat, drought, and salinity, indicating their potential roles in enhancing stress tolerance in rice. Significantly, this study delves into the previously unexplored role of *OsPR1* genes in responding to Brassinosteroid (BR) and heat stress (HS) treatments, confirming their involvement in stress responses through qRT-PCR analysis. We found that seven genes were upregulated by EBR treatment. During heat stress (HS), six and seven genes were upregulated at 1hand 4h HS, respectively. The remaining genes *OsPR1-22* and *OsPR1-75* were upregulated at 1h but downregulated at 4h HS and under EBR treatment. In contrast, *OsPR1-76* was upregulated at both 1h and 4h HS, but downregulated under EBR treatment. Promoters of *PR1* genes in rice and other crops are rich in transcription factor binding sites (TFBSs) and feature a conserved Cysteine-rich secretory protein (SCP or CAP) motif. This study advances our understanding of *PR1* gene regulation and its potential to enhance stress tolerance in rice.

## Introduction

Plants, despite their immobility, constantly face the threat of numerous invading phytopathogens and abiotic stresses. However, their ability to successfully survive these stresses demonstrates their resilience to environmental challenges (Mazumder et al., 2013; Akbudak et al., 2020; Anuradha et al., 2022). Several studies have shown that complex defense signaling pathways are activated in plants to protect them against these environmental stresses (Kundu et al., 2013; Mazumder et al., 2013; Sahni et al., 2016). The interactions between hosts and pathogens or the environment, which ultimately determine the resistance or susceptibility of host plants, have been studied for decades (Timilsina et al., 2020). These interactions, from the early recognition of stress stimuli to the activation of defense responses, follow a well-organized sequence (Prasad et al., 2009; Kundu et al., 2013; Sahni et al., 2016). Defense responses are triggered by various factors, including the generation of reactive oxygen species (ROS), the induction of the hypersensitive response (HR), the interplay of phytohormones, and the synthesis of pathogenesis-related (PR) proteins (Kundu et al., 2013; Mazumder et al., 2013). PR proteins serve as key modulators in plants, accumulating in response to both biotic and abiotic stresses. Low molecular weight PR proteins encompass a diverse array of functions, including roles as transcription factors (TFs) and metabolism-promoting enzymes (Van Loon and Van Strien, 1999; Graham et al., 2003; Hegde and Keshgond, 2013; Jiang et al., 2015). PR1a was the first isolated protein from TMV-infected tobacco leaves (Van Loon and Van Strien, 1999). The expression of *PR1* is induced by a variety of biotic and abiotic stress conditions in *Musa* species and Duram wheat (Akbudak et al., 2020; Anuradha et al., 2022; Zribi et al., 2023) and it serves as an important marker for systemic acquired resistance (SAR). In rice, out of 32 predicted *OsPR1* genes (van Loon et al., 2006), 12 are well characterized for disease resistance in rice (Mitsuhara et al., 2008; Prasad et al., 2009). The functional characteristics of these 12 *OsPR1* genes were performed after treatment with *Xanthomonas oryzae*, *Maganaporthe oryzae*, wounding, SA, JA, and ethylene (Mitsuhara et al., 2008). However, the role of brassinosteroids (BRs) in regulating the 12 *OsPR1* genes has not been studied, despite the fact that BRs are known to regulate numerous genes involved in stress resistance and *PR1*-mediated resistance in Arabidopsis (Zhang et al., 2015). Brassinosteroid (BR) play a significant role in enhancing both abiotic and biotic stress tolerance in various crops and have crosstalk with other defense hormones (Divi et al., 2010; Sahni et al., 2016). BRs have been shown to enhance SA-mediated defense responses. For instance, BRs can amplify the expression of SA-responsive genes and improve resistance against biotrophic pathogens (Divi et al., 2010). *PR1* genes are involved in addressing various stressors, such as salinity and water deficiency, in rice (Kothari et al., 2016) and *Arabidopsis* (Liu et al., 2013). However, there is a surprising lack of research on the effects of heat stress on rice, despite its significant impact on growth, development, yield, and susceptibility to pathogens (Ali et al., 2018a).

Despite significant interest over several decades, the precise roles and functions of all the PR1 genes in plant defense remain poorly understood. To address this knowledge gap, we selected 11 OsPR1 proteins based on the presence of a 10-amino-acid-long conserved sequence (WCCHGCCCYP), which is unique to OsPR1 proteins (Mitsuhara et al., 2008). These 11 *OsPR1* genes, whose responses to both biotic and abiotic stresses have not been explored to date, were chosen for functional characterization. For this purpose, we conducted treatments on rice plants involving wounding, heat stress (HS), as well as the application of two defense hormones: salicylic acid (SA) and brassinosteroid (BR). An important characteristic of pathogen-inducible promoters is their rapid activation in response to multiple phytopathogens. As a result of their interaction with signaling molecules of defense such as SA, jasmonic acid (JA), and ethylene (ET), pathogen-inducible promoters typically contain many potential cis-regulatory elements (Mazarei et al., 2008). Despite this, little is known about the molecular mechanisms governing *PR* gene expression. According to Lodhi et al. (2008), there is a relationship between tobacco *PR1* expression and promoter sequence architecture, nucleosome positioning, and targeting of transcription factors. To elucidate plant defense mechanisms, it is crucial to examine the regulation of the PR1 gene. Plant defense responses are modulated by transcription factors (Liu et al., 2022) interacting with cis-acting regulatory elements (5–20 bp) specific to certain genes (Kaur and Pati, 2016). Therefore, we conducted a comprehensive in silico study of the promoter sequence of *OsPR1* genes to gain insights into the regulation of gene expression by transcription factor binding sites (TFBSs). Furthermore, we employed a similar methodology to identify *PR1* genes in *Brachypodium distachyon, Hordeum vulgare, Brassica rapa*, and *Zea mays*, focusing on the presence of a 10-amino-acid-long conserved sequence. Recent advancements in techniques such as RNAi and RNA-Seq analysis have made it possible to identify and explore the promoter regions of target genes, though these methods are often cost-intensive. Consequently, computational methods are increasingly utilized to discover cis-elements in various promoter regions that govern gene expression (Kaur et al., 2017). The PlantCARE software used for examine the cis-acting regulatory elements (CAREs) within the promoter sequences of PR genes, along with an analysis of their effects, will enhance our comprehension of *PR* gene regulation. A deeper understanding of trans-acting elements can also improve our ability to manipulate gene expression in a desired manner, offering new avenues for applying plant genetic engineering to safeguard crops against various environmental stresses.

## Materials and methods

### Experimental materials

In this study, rice seeds (*Oryza sativa* cv. Rajendra Kasturi) were obtained from Bihar Agricultural University, Sabour, Bhagalpur. Plants were grown up to the four-leaf stage and used for salicylic acid (SA) and wounding treatments under greenhouse conditions. We have taken three biological replicates for both mock and treatment conditions. Each replicate consisted of four individual plants. For 2, 4-epi-brassinolide (EBR) and heat stress (HS) treatments, surface-sterilized seeds (Prasad et al., 2016) were grown on Murashige and Skoog (MS) medium (HiMedia) in magenta boxes (Tarson, India) for 15 days at 25°C under a 14/10 h light/dark cycle in a growth chamber. Rajendra Kasturi is a short-grained, high-yielding aromatic cultivar but is very prone to several abiotic and biotic stresses, reducing its productivity (Kumar et al., 2020).

### Treatments with phytohormones

Twenty-one-day-old rice plants were treated with 3 mM sodium salicylate. To prepare the working solution, a 1 M stock solution of sodium salicylate was initially prepared by dissolving the required amount of sodium salicylate in absolute ethanol. The volume was then adjusted to the desired final volume with distilled water. To prepare the 2, 4-epi-brassinolide (EBR) stock solution, a 100 mM/mL EBR solution was dissolved in absolute ethanol, and a 100 µM/mL working solution was prepared from this stock. For the experiment, a final concentration of 1 µM EBR was used, and for the mock treatment, 0.02% ethanol (the solvent used for EBR) was added to the MS media in test tubes. For the preparation of MS media, MS salts and vitamins (HiMedia) were added at a concentration of 4.42 g/L. Fe-EDTA (200X) was prepared by dissolving 557 mg FeSO_4_•7H_2_O and 745 mg Na_2_EDTA, and 5 mL/L of this solution was added to the MS media. Additionally, 7.5 g/L of Clarigel (HiMedia) was included, and the pH of the media was adjusted to 5.8.

### Pathogen treatment

*X. oryzae* pv. *oryzae* (NCBI GenBank: MH986180) was used for the experiment, following the leaf clipping method as previously described (Kumari et al., 2020, 2022). The 21 day old rice leaves were clipped with scissors dipped in a bacterial suspension (1 × 10^8-9^ cfu/mL) in saline (0.9%) with 0.05% Triton-X-100, and mock treatments used sterile water with 0.05% Triton-X-100 (Kumari et al., 2020, 2022).

### Heat stress treatment

For HS treatment, 15-day-old rice seedlings grown on MS media were exposed to 42°C for 1 hour and 4 hours, then returned to the growth chamber set to 20°C. Untreated seedlings served as controls.

### Phylogenetic analysis of *PR*1 genes

To comprehensively investigate the presence of PR1 proteins in *B. distachyon*, *H. vulgare*, *B. rapa*, *Z. mays*, and *O. sativa*, we conducted BLASTP and HMMER (HMMER 3.1b2; http://hmmer.org/; accessed on 29 April 2020) searches as described previously (Prakash et al., 2017). All the twelve OsPR1 protein sequences were individually subjected for similarity searches against *B. distachyon, H. vulgare, B. rapa*, and *Z. mays* using BLASTP searches with these sequences as queries (https://www.ncbi.nlm.nih.gov/) (Agarwala et al., 2018) (accessed on 12 May 2020). The top ten hits from each plant species were selected and combined. Through manual curation, redundant, incomplete, and closely similar entries in terms of amino acid sequences were eliminated. Additionally, we conducted multiple sequence alignments (MSA) of all the newly identified putative PR proteins along with OsPR1s to verify the presence of a conserved 10-amino-acid-long sequence [W(10x)C(8x)C(x)H(12x)GC(4x)C(9x)C(1x)Y(10x)P]. The conservation of the 10 amino acids is a typical characteristic of PR1 proteins (Mitsuhara et al., 2008). Finally, eighty one putative PR1 sequences were used for phylogenetic tree construction. using the neighbor-joining (NJ) method with bootstrapping (1000 replicates) in the MEGA 11 Program (http://www.megasoftware.net; Analysis version 1.01; accessed on 20 August 2020) (Tamura et al., 2021).

### *In silico* analysis of putative domains, subcellular localization and chromosomal position of protein

All PR1 proteins were subjected for domain and subcellular localization studies. The domain prediction was performed using the Pfam tool (Pfam 37.0; http://pfam.xfam.org/search/sequence; accessed on 10 September 2021) (Mistry et al., 2021) and subcellular localization was predicted using the Balanced Subcellular Localization Predictor tool (BaCelLo) (http://gpcr.biocomp.unibo.it/bacello/info.htm; accessed on 10 September 2021) (Pierleoni et al., 2006). Chromosomal positions of *PR1* genes were studied using Phytozome v12.1 database (https://phytozome.jgi.doe.gov/pz/portal.html; Analysis version v12.1; accessed on 20 August 2020). Chromosome maps of *O. sativa PR1* gene were constructed using Chromosome Map Tools Oryza base (http://viewer.shigen.info/oryzavw/maptool/MapTool.do; accessed on 27 August 2022).

### Analysis of cis-regulatory elements

The Rice Annotation Project Database (RAP-DB), (http://rapdb.dna.affrc.go.jp/tools/dump; accessed on 21 January 2021) (Sakai et al., 2013) was utilized to obtain 1000 base pair upstream sequences of 23 *OsPR1* genes. These retrieved sequences were subsequently analyzed using PlantPan3, (http://plantpan3.itps.ncku.edu.tw/; accessed on 21 January 2021) (Chow et al., 2016), for the identification of transcription factor binding sites (TFBS), and PlantCARE, (http://bioinformatics.psb.ugent.be/webtools/plantcare/html; accessed on 21 January 2021) (Lescot, 2002), for the analysis of cis-regulatory DNA elements in plants.

In addition, we retrieved 1000 base pair upstream sequences for newly identified *PR1* genes in various plant species using Phytozome 12 (https://phytozome.jgi.doe.gov/pz/portal.html; Analysis version v12.1; accessed on 30 January 2021) (Goodstein et al., 2012). TFBSs and cis-regulatory DNA elements within these sequences were analyzed using the same approach as described above. A detailed flow chart for the identification of cis-regulatory sequences in rice and other plants is depicted in **Supplementary Figure 1**.

### RNA isolation and quantitative real-time RT-PCR

The relative expression of 11 *OsPR1* genes, including the reference gene *OsPR1a*, in rice seedlings treated with salicylic acid (SA), epibrassinolide (EBR), mechanical wounding, and heat stress (HS) was measured by qRT-PCR analysis. Total RNA was extracted from frozen plant tissues using Promega’s SV Total RNA Isolation System following the manufacturer’s protocol (Promega, Madison, WI). For cDNA synthesis, 1µg of total RNA was reverse transcribed using random hexamer primers according to manufacturer’s protocol (Promega, Madison, WI). The resulting cDNA was diluted in nucleases free water (1:5) and used for qRT-PCR (Kumari et al., 2022; Chaudhary et al., 2024). The qRT-PCR was carried out in a Light Cycler system (Applied Biosystems) using SYBR Green dye. Each reaction mixture (10 µL) contained 5 µL of SYBR Green dye (2×) (Promega, Madison, WI), 0.5 µL of forward/reverse gene-specific primers (10 µM), and 1 µL of diluted cDNA.

Three independent biological replicates as well as two technical repeats were conducted for each qRT-PCR analysis. The qRT-PCR conditions included an initial denaturation at 95°C for 2 minutes, followed by 40 cycles of amplification consisting of denaturation, annealing, and extension at 95°C, 53°C, and 72°C for 20 s, 30 s, and 30 s, respectively. The specificity of amplification was confirmed by melting curve analysis. ACTIN was used to normalize the expression data of *OsPR1* genes. The fold-change in expression levels of genes was estimated using the 2^-ΔΔCt^ method as described previously (Livak and Schmittgen, 2001).

### Statistical analysis

Gene expression data was statistically analyzed using the computer software SPSS. Significance of differences were analyzed by one-way analysis of variance (ANOVA).

## Results

### Identification of PR1 proteins in important crops

The BLASTP analysis yielded 82 PR1 proteins using OsPR1 as the query sequence, and the HMMER search results identified 145 PR1 proteins. To ensure accuracy, we manually excluded PR1 proteins that were common between the BLASTP and HMMER searches. The initial extensive list of 227 PR1 proteins was refined to 81 proteins based on the presence of a conserved 10-amino-acid sequence [W(10x)C(8x)C(x)H(12x)GC(4x)C(9x)C(1x)Y(10x)P]. Among the 81 PR1 proteins analyzed, 23 were identified in *Oryza sativa*, while putative orthologs were found in *Brachypodium distachyon, Hordeum vulgare, Brassica rapa*, and *Zea mays*, totaling 58 proteins across these species. In this study, 23 *OsPR1* genes were selected for phylogenetic analysis, with a subset of 11 *OsPR1* genes chosen specifically for expression analysis experiments.

Each gene sequence was designated according to its chromosomal position and gene order (**Tables 1**, **2**). For example, *OsPR1-21* (*LOC_OS02G54530.1*) is located on chromosome 2, while *OsPR1-61* (*LOC_OS06G24290.1*) is situated on chromosome 6. Notably, chromosome 7 houses a cluster of eight *PR1* genes, namely *OsPR1-71, -72, -73, -74, -75, -76, -77*, and -*78*. This cluster is organized in a consecutive arrangement from the 5’ end to the 3’ end, all aligned in the sense orientation, as described by Mitsuhara et al. (2008).

**Table 1:** Description of Rice Pathogenesis related (*OsPR*1) genes and their promoter regions.

Protein ID	MSU (LOC_OsID)	BaCello Protein Localization	SCP Domain (aa)	Peptide sequence (aa)	No of exon, Intron	Chromosome	Genomic sequence	CDS Sequence
LOC_OS01G28450.1 (OsPR1#011)	LOC_Os01g28450.1	Secretory	24-160 (136)	165,	1, 0	1	829	495
LOC_OS01G28500.1 (OsPR1# 012)	LOC_Os01g28500.1	Secretory	29-163 (134)	168	1, 0	1	766	504
LOC_OS02G54540.1 (OsPR1# 021)	LOC_Os02g54540.1	Secretory	38-168 (130)	173	1, 0	2	519	519
LOC_OS02G54560.1 (OsPR1# 022)	LOC_Os02g54560.1	Secretory	58-195 (137)	200	1, 0	2	1614	600
LOC_OS05G51660.1 (OsPR1# 051)	LOC_Os05g51660.1	Secretory	54-186 (132)	199	1, 0	5	1077	597
LOC_OS05G51680.1 (OsPR1# 052)	LOC_Os05g51680.1	Secretory	113-242 (129)	333	3, 2	5	3362	999
LOC_OS07G03279.1 (OsPR1# 071)	LOC_Os07g03279.1	Secretory	30-167 (137)	180	1, 0	7	773	540
LOC_OS07G03580.1 (OsPR1# 072)	LOC_Os07g03580.1	Secretory	28-168 (140)	173	1, 0	7	714	519
LOC_OS07G03590.1 (OsPR1# 073)	LOC_Os07g03590.1	Secretory	28-165 (137)	170	1, 0	7	1260	510
LOC_OS07G03710.1 (OsPR1# 074)	LOC_Os07g03710.1	Secretory	26-164 (138)	169	1, 0	7	934	507
LOC_OS10G11500.1 (OsPR1# 101)	LOC_Os10g11500.1	Secretory	30-164 (134)	177	1, 0	10	739	531
LOC_OS12G43700.1 (OsPR1# 121)	LOC_Os12g43700.1	Secretory	282-414 (132)	419	2, 1	12	1838	1257
LOC_OS02G54530.1 (OsPR1-21)	LOC_Os02g54530.1	Secretory	35-174 (139)	179	1, 0	2	571	537
LOC_OS02G54570.1 (OsPR1-22)	LOC_Os02g54570.1	Secretory	40-174 (134)	179	1, 0	2	537	537
LOC_OS06G24290.1 (OsPR1-61)	LOC_Os06g24290.1	Secretory	27-160 (133)	176	1, 0	6	528	528
LOC_OS07G03409.1 (OsPR1-71)	LOC_Os07g03409.1	Secretory	27-168 (141)	173	1, 0	7	519	519
LOC_OS07G03600.1 (OsPR1-72)	LOC_Os07g03600.1	Secretory	27-172 (145)	177	1, 0	7	758	531
LOC_OS07G03680.1 (OsPR1-73)	LOC_Os07g03680.1	Secretory	33-178 (145)	183	1, 0	7	549	549
LOC_OS07G03690.1 (OsPR1-74)	LOC_Os07g03690.1	Secretory	30-168 (138)	173	1, 0	7	519	519
LOC_OS07G03730.1 (OsPR1-75)	LOC_Os07g03730.1	Secretory	23-161(138)	166	1, 0	7	977	498
LOC_OS07G03740.1 (OsPR1-76)	LOC_Os07g03740.1	Nuclease	231-361 (130)	366	6, 5	7	7029	1098
LOC_OS07G14030.1 (OsPR1-77)	LOC_Os07g14030.1	Secretory	35-172 (137)	177	1, 0	7	531	531
LOC_OS07G14070.1 (OsPR1-78)	LOC_Os07g14070.1	Secretory	160-297 (137)	302	3, 2	7	2199	906

**Table 2:** Description of pathogenesis related (PR1) genes in *Z. mays*, *Brachypodium distachyon*, *Hordeum vulgare*, and *Brassica rapa*.

Protein Locus ID/Proposed ID	Phytozome ID	Strand	Protein Localization	Genomic sequence	CDS Sequence	Peptide sequence (aa)	SCP Domain	CN
*Z. mays*								
*ONM08903.1 (ZmPR1-11)*	Zm00001d033902/ GRMZM2G481194	Reverse	Secretory	1141	612	203	65-199 (134)	1
*ONL98986.1 (ZmPR1-12)*	Zm00001d029558/GRMZM2G304442	Reverse	Secretory	877	540	179	33-167 (134)	1
*ONM32708.1 (ZmPR1-31)*	Zm00001d041230/ GRMZM2G163099	Reverse	Secretory	899	804	207	132-263 (131)	3
*AQK75611 (ZmPR1-51)*	Zm00001d018321/AC211357.4_FG002	Forward	Secretory	510	510	170	37-172 (135)	5
*AQK75612.1 (ZmPR1-52)*	Zm00001d018322/GRMZM5G852886	Forward	Chloroplast	717	528	175	38-171 (133)	5
*AQK75613.1 (ZmPR1-53)*	Zm00001d018323/GRMZM2G008406	Forward	Secretory	903	621	206	66-202 (136)	5
*ONM51204.1 (ZmPR1-71)*	Zm00001d018737/ GRMZM2G437187	Forward	Secretory	686	510	169	31-165 (134)	7
*ONM51205.1 (ZmPR1-72)*	Zm00001d018738/GRMZM2G465226	Forward	Secretory	1063	492	163	25-159 (134)	7
*ONM52792.1 (ZmPR1-73)*	Zm00001d019364/GRMZM2G053493	Forward	Secretory	777	516	171	31-167 (136)	7
*XP_008651988.1 (ZmPR1-74)*	Zm00001d018734/ GRMZM2G456997	Forward	Secretory	813	504	167	27-163 (136)	7
*ACJ62559.1 (ZmPR1-81)*	Zm00001d009296/AC205274.3_FG001	Reverse	Secretory	504	504	167	29-163 (134)	8
*B. distachyon*								
*XP_003561249.1 (BdPR1-11)*	Bradi1g12360.1/ BdiBd21-3.1G0165000	Forward	Secretory	586	516	171	25-161 (136)	1
*XP_003557605.1 (BdPR1-12)*	Bradi1g57540.1/ BdiBd21-3.1G0772000	Forward	Secretory	789	516	171	33-167 (134)	1
*KQK20923.1 (BdPR1-13)*	Bradi1g57580.1/ BdiBd21-3.1G0772600	Forward	Secretory	843	498	165	26-161 (135)	1
*KQK20924.1 (BdPR1-14)*	Bradi1g57590.1/ BdiBd21-3.1G0772700	Forward	Secretory	976	501	164	28-160 (132)	1
*KQK04554.1 (BdPR1-21)*	Bradi2g14240.1/ BdiBd21-3.2G0188100	Forward	Secretory	711	633	210	72-205 (133)	2
*XP_010230947.3 (BdPR1-22)*	Bradi2g14256/ BdiBd21-3.2G0188300	Reverse	Secretory	1163	660	219	83-218 (135)	2
*XP_003572883.1 (BdPR1-31)*	Bradi3g53630.1/ BdiBd21-3.3G0709300	Reverse	Secretory	1002	543	180	42-176 (134)	3
*XP_003572884.1 (BdPR1-32)*	Bradi3g53637.1/ BdiBd21-3.3G0709400	revere	Secretory	1771	540	178	35-174 (139)	3
*XP_014756083.2 (BdPR1-33)*	Bradi3g60230.1/ BdiBd21-3.3G0792800	Reverse	Secretory	618	618	205	60-201 (141)	3
*KQK02081.1 (BdPR1-34)*	Bradi3g60260.2/ BdiBd21-3.3G0793200	Forward	Secretory	609	609	202	59-198 (139)	3
*XP_003579097.2 (BdPR1-41)*	Bradi4g00865.1/ BdiBd21-3.4G0008700	Forward	Secretory	1728	1014	332	196-328 (132)	4
*H. vulgare*								
*KAE8766408.1 (HvPR1-11)*	HORVU1Hr1G095440.1	Reverse	Secretory	1207	762	253	108-241 (133)	1
*(HvPR1-31)*	HORVU3Hr1G051110.1	Forward	Secretory	709	447	148	14-122 (108)	3
*(HvPR1-51)*	HORVU5Hr1G001690.1	Forward	Secretory	1720	1140	379	251-383 (132)	5
*(HvPR1-52)*	HORVU5Hr1G001720.1	Reverse	Nuclease	1691	729	142	12-138 (126)	5
*KAE8807165.1 (HvPR1-53)*	HORVU5Hr1G055950.1	Forward	Secretory	753	513	164	26-160 (134)	5
*KAE8810549.1 (HvPR1-54)*	HORVU5Hr1G106010.1	Forward	Secretory	1401	501	166	25-162 (137)	5
*KAE8805026.1 (HvPR1-55)*	HORVU5Hr1G106020.1	Forward	Secretory	679	504	167	25-163 (138)	5
*KAE8806578.1 (HvPR1-61)*	HORVU6Hr1G083390.1	Reverse	Secretory	1109	642	213	72-209 (137)	6
*(HvPR1-71)*	HORVU7Hr1G022230.1	Reverse	Secretory	863	504	168	31-164 (133)	7
*Q05968.1 (HvPR1-72)*	HORVU7Hr1G033530.1	Forward	Secretory	959	579	164	26-160 (134)	7
*BAK01044.1 (HvPR1-73)*	HORVU7Hr1G040730.1	Reverse	Secretory	1306	519	172	27-160 (133)	7
*KAE8813015.1 (HvPR1-74)*	HORVU7Hr1G040740.1	Forward	Secretory	133617	360	158	11-144 (133)	7
*B. rapa*								
*RID78779.1 (BrPR1-11)*	Brara.A01572.1	Forward	Secretory	852	609	202	62-198 (136)	1
*RID78780.1 (BrPR1-12)*	Brara.A01573.1	Reverse	Secretory	1459	630	209	73-205 (132)	1
*XP_009127912.1 (BrPR1-13)*	Brara.A00713.1	Reverse	Secretory	507	507	168	32-164 (132)	1
*RID77527 (BrPR1-14)*	Brara.A00432.1	Reverse	Secretory	696	519	172	37-168 (131)	1
*RID77528 (BrPR1-15)*	Brara.A00433.1	Reverse	Secretory	525	525	174	28-161 (133)	1
*VDC86616.1 (BrPR1-21)*	Brara.B01198.1	Reverse	Secretory	1324	624	207	70-203 (133)	2
*BR_RID69062 (BrPR1-31)*	Brara.C01183.1	Reverse	Nucleus	1730	681	226	89-222 (133)	3
*VDC81544.1 (BrPR1-32)*	Brara.C03265.1	Reverse	Secretory	573	573	192	50-154 (104)	3
*XP_009135645.1 (BrPR1-33)*	Brara.C03754.1	Reverse	Secretory	507	507	168	31-164 (133)	3
*XP_009136246.1 (BrPR1-34)*	Brara.C04098.1	Reverse	Secretory	483	483	160	27-156 (129)	3
*AAT46023.1 (BrPR1-35)*	Brara.C04099.1	Reverse	Secretory	486	486	161	28-157 (129)	3
*BR_RIA05031 (BrPR1-36)*	Brara.K00636.1	Reverse	Secretory	489	489	162	27-158 (131)	3
*BR_RIA05035 (BrPR1-37)*	Brara.K00640.1	Reverse	Secretory	492	492	163	28-159 (131)	3
*XP_009137911.1 (BrPR1-38)*	Brara.K00836.1	Reverse	Secretory	501	501	166	30-162 (132)	3
*BR_RID56911 (BrPR1-61)*	Brara.F00326.1	Reverse	Chloroplast	576	489	162	26-158 (132)	6
*BR_RID52650 (BrPR1-71)*	Brara.G00101.1	Reverse	Secretory	624	537	178	34-168 (134)	7
*BR_RID52651 (BrPR1-72)*	Brara.G00102.1	Reverse	Secretory	660	522	173	33-166 (133)	7
*BR_RID50524 (BrPR1-81)*	Brara.H01250.1	Reverse	Secretory	513	513	170	28-166 (138)	8
*VDC83896.1 (BrPR1-82)*	Brara.H01347.1	Forward	Chloroplast	1288	432	181	45-177 (132)	8
*BR_RID50861 (BrPR1-83)*	Brara.H01563.1	Reverse	Secretory	558	558	185	45-181 (136)	8
*BR_RID44342 (BrPR1-91)*	Brara.I01145.1	Reverse	Secretory	522	522	173	33-166 (133)	9
*BR_RID49143 (BrPR1-92)*	Brara.I05604.1	Forward	Chloroplast	1017	732	243	85-217 (132)	9
*VDD16626.1 (BrPR1-101)*	Brara.J00054.1	Reverse	Chloroplast	720	720	239	81-213 (132)	10
*BR_RID43023 (BrPR1-102)*	Brara.J02861.1	Reverse	Secretory	561	561	186	44-178 (134)	10

### Phylogenetic analysis of PR1 proteins

**Figure 1:**
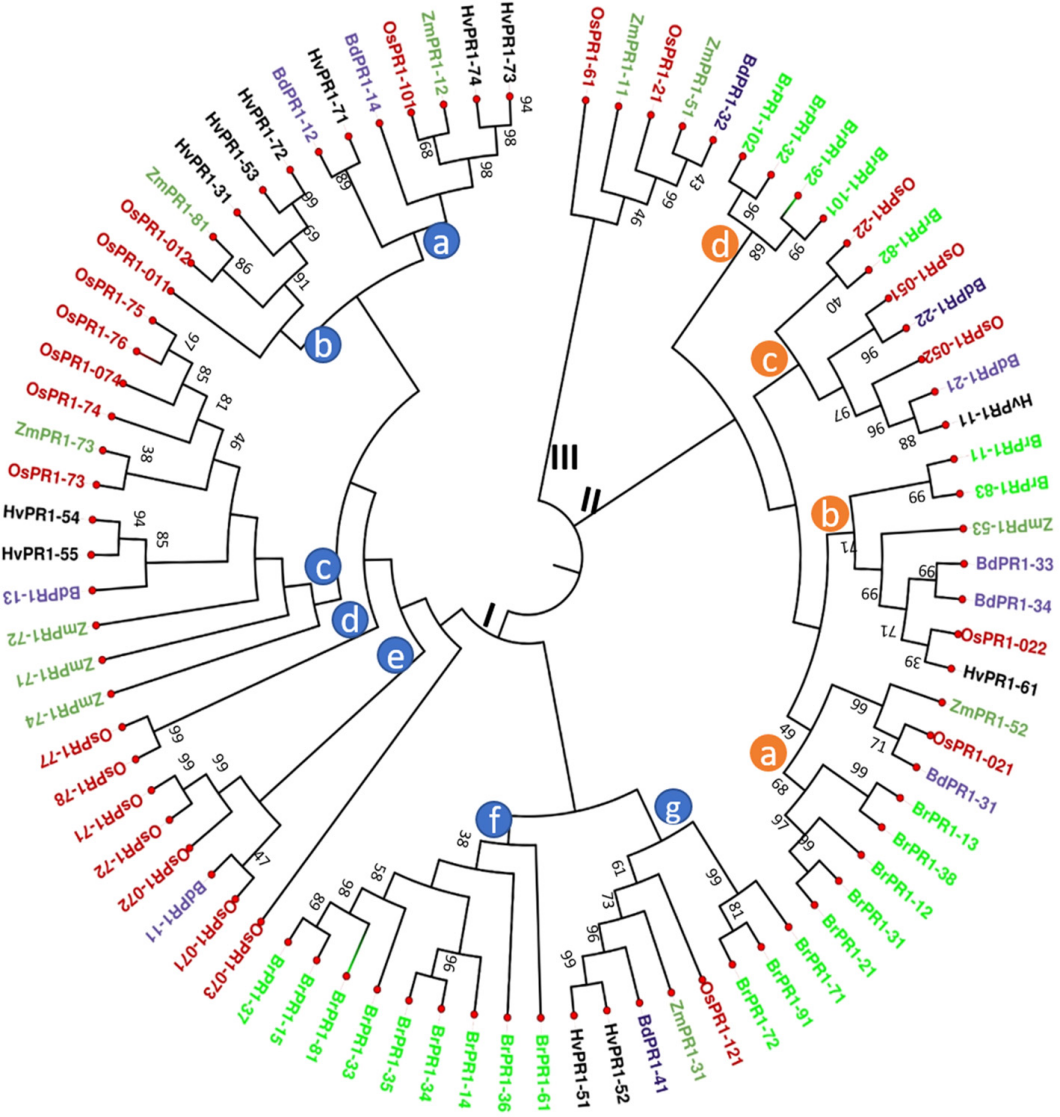
Phylogenetic tree analysis of PR1 protein families. The phylogenetic relationship of OsPR1 proteins with homologs from other important plant species was constructed using the MEGA 11 program, after aligning the protein sequences with MUSCLE. The protein sequences were obtained from Phytozome12. Different PR1 proteins are highlighted in various color codes for easy identification: OsPR1 (*Oryza sativa*) in red, ZmPR1 (*Zea mays*) in green, BdPR1 (*Brachypodium distachyon*) in blue, HvPR1 (*Hordeum vulgare*) in black, and BrPR1 (*Brassica rapa*) in fluorescent green. The phylogenetic tree analysis revealed distinct clusters, denoted as Cluster I, Cluster II, and Cluster III. Cluster I can be further divided into several subgroups, represented as clade groups a, b, c, d, e, f, and g, which are identified with blue circles on the phylogenetic tree. Notably, Cluster I contains the highest number of PR1 proteins, totaling 50. Cluster II consists of subgroups a, b, c, and d, which are indicated by brown circles on the phylogenetic tree.

The neighbor-joining (N-J) method of MEGA 11 Program (Tamura et al., 2021) was used to create a phylogenetic tree to compare OsPR1 and its potential homologs in both monocot and dicot plants. The analysis resulted in the formation of three main clusters: Cluster I, II, and III (**Figure 1**). Further, these clusters were divided into groups (a-f) based on clade division.

Cluster I contains the largest number (50) of PR1 proteins, including Group C proteins such as OsPR1a and OsPR1-74. Previous reports have used OsPR1a as a versatile marker for both biotic and abiotic studies in rice (Yan et al., 2022). Within Cluster I, OsPR1-011 (OsPR1b) is positioned closer to OsPR1-75. Notably, OsPR1-011 demonstrates upregulation after salicylic acid (SA) treatment but downregulation following wounding (Mitsuhara et al., 2008). Similarly, our study observed the same trend for OsPR1-75. In Cluster I, the Group a, b, and c proteins are clustered together, containing ZmPR1-12 and OsPR1-101, ZmPR1-81 and OsPR1-012, and ZmPR1-73 and OsPR1-73 within the same clade BdPR1-14 (Bradi1g57590) and OsPR1-101. Notably, both of these genes were observed to be upregulated following SA treatment (Mitsuhara et al., 2008; Kakei et al., 2015). Cluster II represents the second largest group of PR1 proteins (**Figure 1**). In Groups B and C of Cluster II, HvPR1-61 and BrPR1-82 along with OsPR1-022 and OsPR1-22, are grouped within the same clade (**Figure 1**).

### *In silico* analysis for putative domain search, subcellular localization of proteins and chromosomal position

All the identified PR1 proteins contain a conserved motif belonging to the cysteine-rich secretory protein (SCP or CAP) domain, which ranges from 104 to 145 amino acids (**Tables 1**, **2**). Subcellular localization predictions indicated that the majority of PR1 proteins are secretory in nature (**Tables 1**, **2**). However, some proteins, such as OsPR1-76, HvPR1-52, HvPR1-53, and HvPR1-73, were predicted to localize in the nucleus. ZmPR1-52 was predicted to localize in the chloroplast.

**Figure 2:**
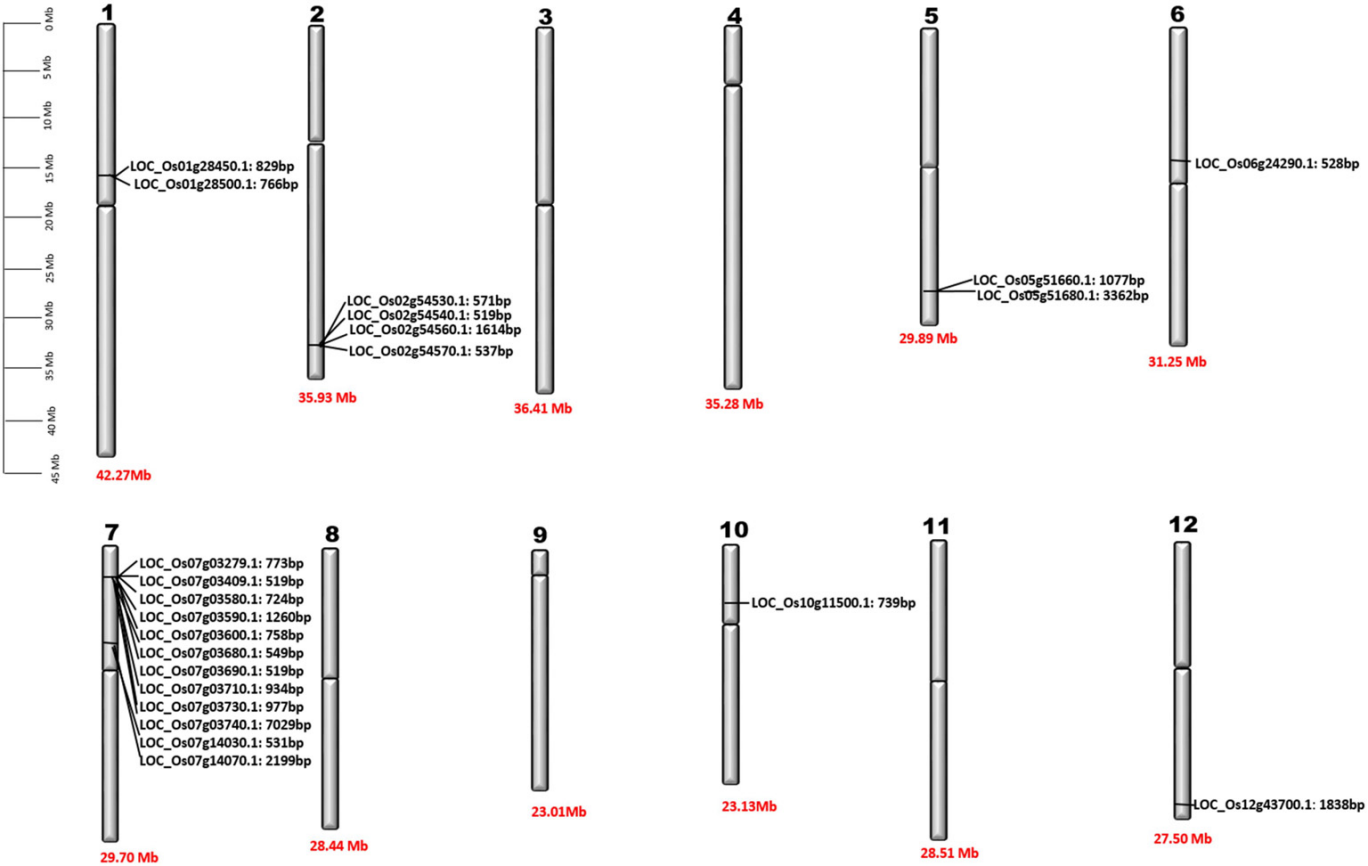
Chromosomal location of *OsPR1* genes. Detailed physical maps of individual chromosomes have been meticulously generated, clearly indicating the specific positions of individual *OsPR1* genes in base pair (bp) units. Furthermore, the length of each chromosome is precisely denoted at the bottom of the graphical representations, expressed in Megabase (Mb) measurements. It highlights an enrichment pattern of *OsPR1* genes on chromosomes 7, 2, 5, 1, 6, 10, and 12, indicating a higher density of these genes in these genomic regions. Interestingly, no *OsPR1* genes were found on chromosomes 3, 4, 8, 9, and 11.

Our findings indicate that *BdPR1, HvPR1, BrPR1, ZmPR1*, and *OsPR1* genes are situated on distinct chromosomes (**Figure 2**). Notably, chromosome 7 exhibited the highest concentration of *PR1* genes, comprising 25.5% of the total. Chromosomes 1 and 3 collectively harbored 16% of the *PR1* genes, while the remaining genes were distributed across other chromosomes (**Tables 1**, **2**; **Figure 2**).

### *OsPR1* gene expression in response to hormonal treatments

Plant hormones, salicylic acid (SA) and brassinosteroids (BR), have been identified as key regulators of the plant defense system (Kumari et al., 2022). While the role of SA in regulating PR1 genes during plant defense is well-established (Takatsuji et al., 2010), the precise impact of BR on *PR1* gene expression is largely unknown. In the present investigation, a total of 11 *OsPR1* genes, whose significant roles had not been previously studied, were meticulously selected for comprehensive expression analysis, along with the *OsPR1a* marker gene included for reference.

**Figure 3:**
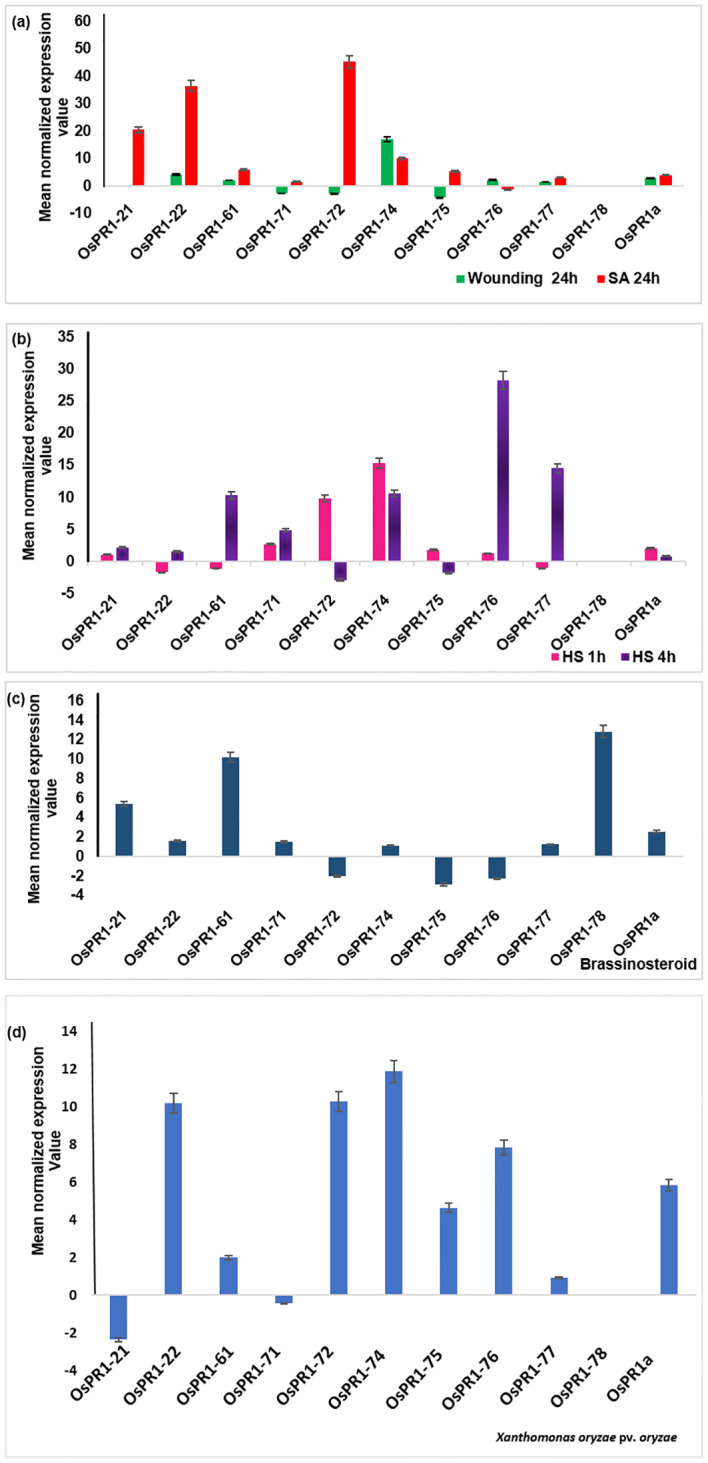
Transcriptional response of 11 *OsPR1* genes to selected treatments salicylic acid, wounding, heat, and 2, 4-epibrassinolide treatments were studied **(A)**. Transcriptional response of 11 *OsPR1* genes to with salicylic acid. Rice seedlings were treated with 3mM salicylic acid and wounded in criss-cross manner to analyze the transcript levels by qRT-PCR at 24h post treatment. **(B)**. Transcriptional response of 11 *OsPR1* genes treated with heat stress. Rice seedling grown in half MS media placed in incubator at 42 °C. **(C)**. Transcriptional response of 11 *OsPR1* genes in rice seedlings treated with 2, 4-epibrassinolide. Rice seedlings were grown for 15 days on MS medium supplemented with EBR. **(D)** Rice seedlings were infected with *Xanthomonas. oryzae* pv. *oryzae*, and the transcript levels of *OsPR1* genes were analyzed using qRT-PCR at 24h post-infection. Relative transcript abundance is expressed as mean normalized expression value relative to the mock treatment. *OsPR1a* expression served as a positive control, indicating successful infection of rice seedlings by salicylic acid. Results are representative of three independent experiments. Error bars represent standard error (SE) of mean for three replicates.

Upon subjecting the rice seedlings to SA treatment, eight genes *OsPR1-21*(~21-fold induction)*, OsPR1-22* (~37 fold induction)*, OsPR1-61* (~6-fold induction)*, OsPR1-*71(~1.5-fold induction)*, OsPR1-72* (~45-fold induction)*, OsPR1-74* (~9.5-fold induction)*, OsPR1-75* (~5.6-fold induction), and *OsPR1-77* (~3.1-fold induction)) showed upregulation, while *OsPR1-76* displayed downregulation. Notably, *OsPR1-73* and *OsPR1-78* were not amplified in uniform manner in treated or mock samples (**Table 3**, **Figure 3A**).

**Table 3:** Summary of gene expression changes after all treatments.

*OsPR1* genes	SA	EBR	Wounding	HS 1h	HS 4h	Xoo
*OsPR1-21*	+++++	+	NA	+	+	–
*OsPR1-22*	6+	+	+	–	+	++
*OsPR1-61*	++	++	+	–	++	+
*OsPR1-71*	+	+	–	+	+	–
*OsPR1-72*	9+	–	–	++	–	++
OsPR1-73*	NA	NA	NA	NA	NA	NA
*OsPR1-74*	++	+	++++	+++	+++	+++
*OsPR1-75*	+	–	–	+	–	+
*OsPR1-76*	–	–	+	+	6+	++
*OsPR1-77*	+	+	+	–	+++	+
*OsPR1-78*	NA	+++	NA	NA	NA	NA
*OsPR1a*	+	+	+	+	+	+

Upregulation fold: 1-5= (+), 5-10= (++), 10-15= (+++), 15-20= (++++) and so on, Downregulation fold: 1-5= (-), NA, Not amplified in uniform manner in all biological repeats.

Following EBR-treated, seven *OsPR1* genes *OsPR1-21, OsPR1-22, OsPR1-61, OsPR1-71, OsPR1-74, OsPR1-77*, and *OsPR1-78* were found to be upregulated whereas three genes *OsPR1-72, OsPR1-75*, and *OsPR1-76* were downregulated. The maximum induction was observed in *OsPR1-78* (~12- fold induction), followed by *OsPR1-*61 (~10-fold induction) and *OsPR1-*21 (~5-fold induction) (**Figure 3C**).

### *OsPR1* gene expression analysis after HS treatment

Upon 4h of HS treatment, *OsPR1-*76 showed the highest upregulation (~28-fold induction) while *OsPR1-77* showed the lowest upregulation (~14 -fold induction). Both *OsPR1-*74 and *OsPR1-*61 showed upregulated to the same level (~10-fold induction) upon 4h of HS. *OsPR1-*72 and *OsPR1-75* genes were downregulated ~2– ~3-fold after 4 h of HS, whereas *OsPR1-*22, *OsPR1-61*, and *OsPR1-*77 were downregulated (~1.5-fold) after 1 h of HS treatment. *OsPR1-*74 showed the highest upregulation (~15-fold induction) after 1 h of HS-treatment, followed by *OsPR1-*72 (~10-fold induction), while the remaining upregulated genes showed upregulation in range of ~ 1-3-fold. Following exposure to HS, *OsPR1-73* and *OsPR1-78* were unamplified (**Figure 3B**).

### *OsPR1* expression in response to wounding and pathogen treatment

The leaves of 21 day old rice seedlings were subjected to precise and effective cross-cross wounding employing sterile scissors as described previously (Mitsuhara et al., 2008). Our result revealed that the maximum expression was observed in *OsPR1-*74 gene (~17-fold induction), followed by the *OsPR1-*22 gene (~4-fold induction). Three genes-*OsPR1-*75 (~4-fold), *OsPR1-*71 (~2.7-fold), and *OsPR1-*72 (~4.3-fold) were found to be downregulated (**Figure 3A**). The responses of all *11 OsPR1* genes to different treatments are summarized in **Table 3**.

Genes that were highly upregulated at 24 hours post-infection with *X. oryzae* pv. *oryzae* included *OsPR1-74* (~12-fold), followed by *OsPR1-72* and *OsPR1-22* (~10-fold), *OsPR1-76* (~7.86-fold), *OsPR1-75* (~4.64-fold), and *OsPR1-61* (~2-fold). In contrast, *OsPR1-73* and *OsPR1-78* did not show consistent amplification across all biological replicates (**Figure 3D**).

### Retrieval of promoter regions and analysis of plant cis-acting regulatory elements and transcription factor binding sites in rice

**Figure 4:**
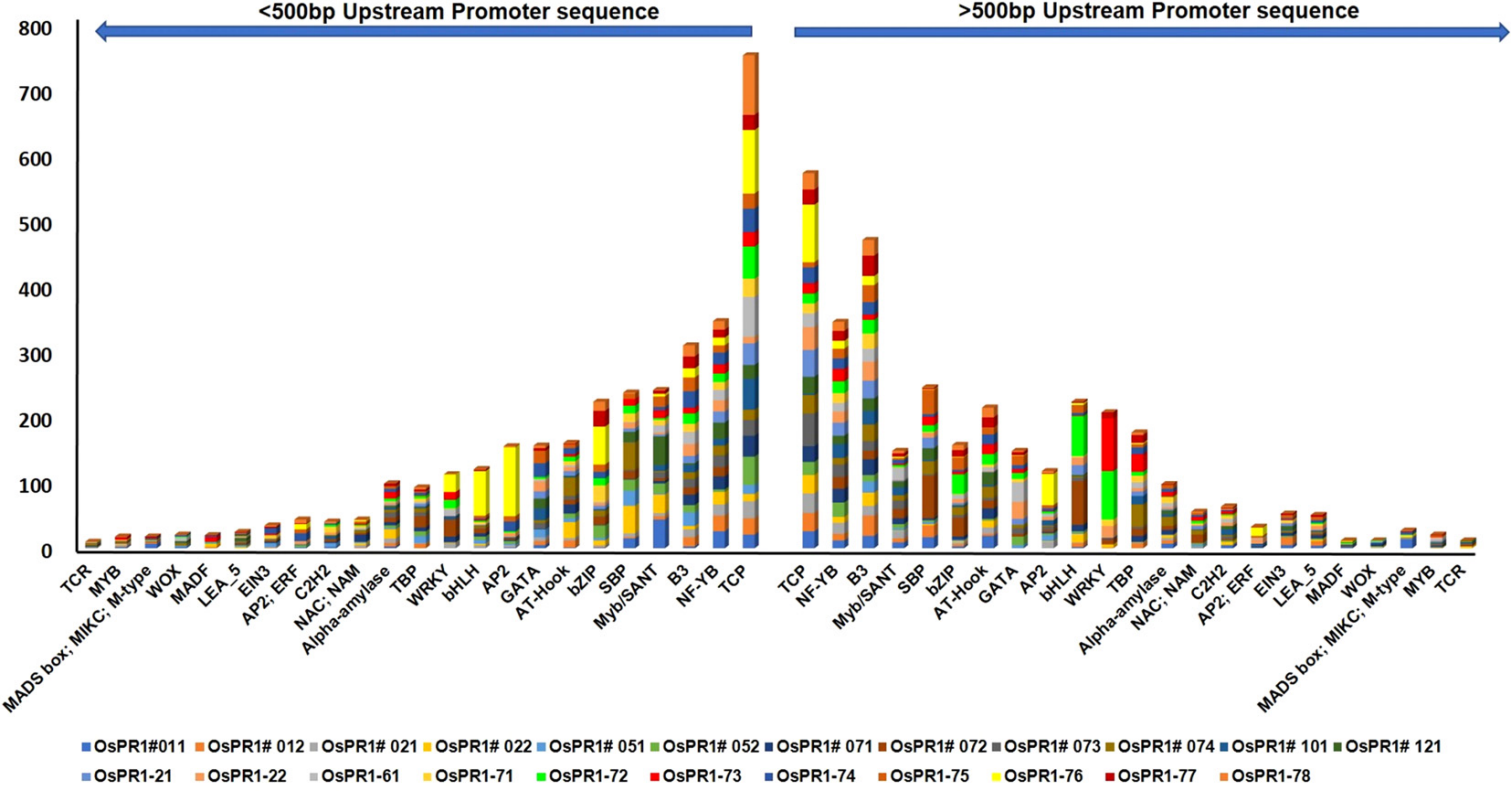
Enrichment of transcription factor binding sites (TFBSs) in *OsPR1* promoter. The abundance of stress and developmental related TFBSs shown in *OsPR1* promoter proximal (<500 bp) and distal (>500 bp) region. TFs is key component in transcription regulation of gene expression during multiple stress.

**Figure 5:**
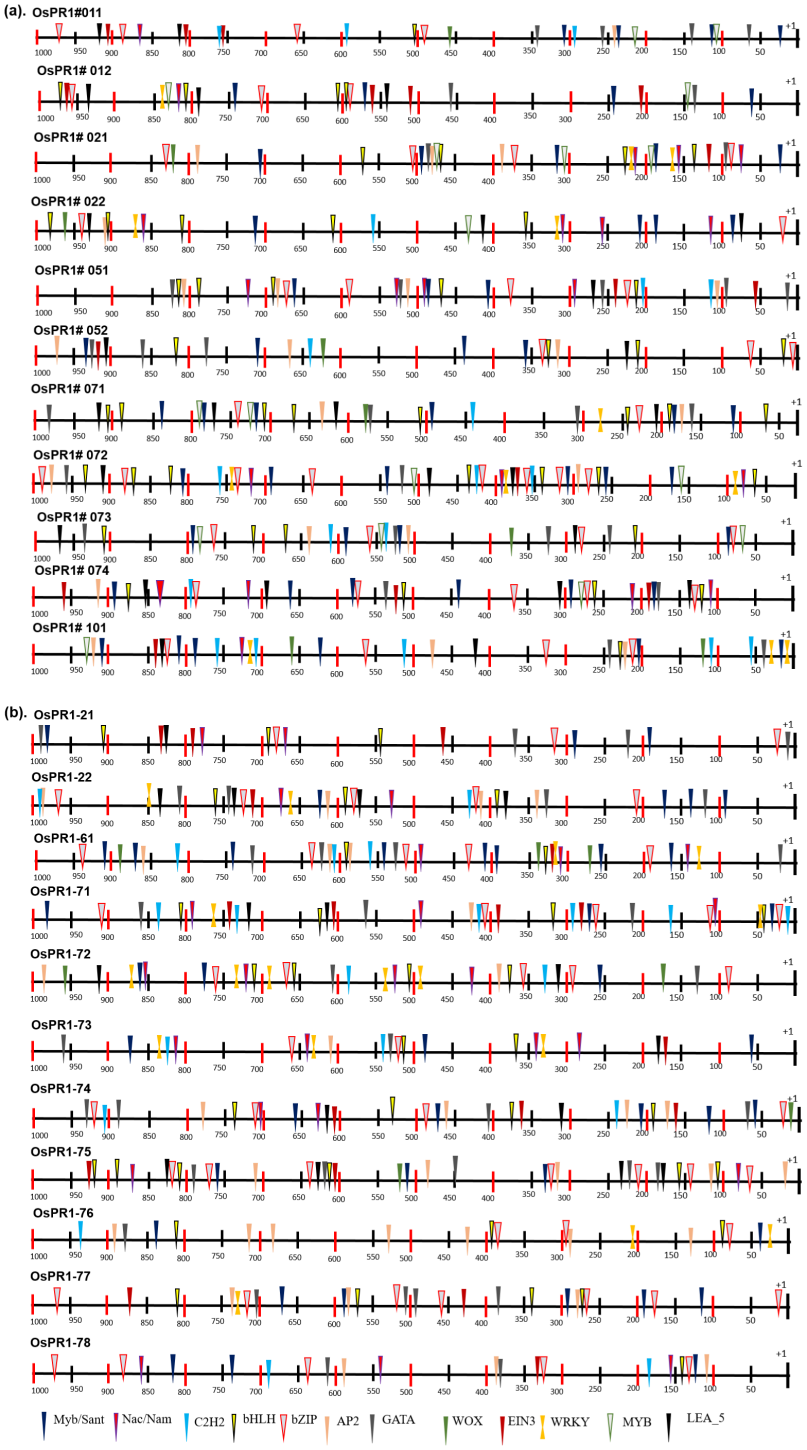
Distribution of transcription factor binding sites (TFBSs) in 1000 bp promoter region of rice *PR1* genes. Different TFBSs denoted by different shapes and color code combination as identified by PlantPan3. Promoter prediction tool. **(A)** Distribution of transcription factor binding sites (TFBSs) of previously reported *OsPR1* (Mitsuhara et al., 2008). **(B)** Distribution of transcription factor binding sites (TFBSs) in currently studied *OsPR1*.

The promoter sequences located up to 1 kilobase (kb) upstream from the translation start site of each *PR* gene in *Oryza sativa* (rice) were meticulously analyzed using PlantCARE and PlantPAN 3 programs. The primary goal was to identify potential plant cis-acting regulatory elements (PCAREs) and transcription factor binding sites (TFBSs) associated with defense responses. A diverse set of important defense-responsive TFBSs, such as bHLH, bZIP, C2H2, EIN3, GATA, LEA, MYB, Myb/SANT, NAC, NAM, and WRKY, were successfully identified in both the positive and negative strands of the promoter sequences of *OsPR1* genes (**Figures 4**, **5**). The analysis further focused on determining the enrichment and distribution of all TFBSs within proximal (<500 bp) and distal (>500 bp) regions of all *OsPR1* genes. Remarkably, it was observed that the majority of these TFBSs were significantly enriched in the proximal upstream regions of most *OsPR1* genes. This finding highlights the potential significance of these regulatory elements in modulating the expression of *OsPR1* genes during defense responses in rice (**Figures 4**, **5**). In our experiment, the highest number of WRKY TFBSs was found in *OsPR1-73* (93); however, this gene was not expressed under any of the treatments. In contrast, a significant enrichment of WRKY TFBSs was observed in *OsPR1-72* (88), which was upregulated 45-fold and 10-fold following SA and heat stress treatments, respectively (**Supplementary Table 2**). This suggests that WRKY TFBSs play a major role in PR1-mediated biotic and abiotic responses. *OsPR1-76* has the highest number of AP2 (175) and bZIP (61) TFBSs (**Supplementary Table 2**) and shows maximum expression during 4 hours of heat stress (28-fold), as well as 1 hour of heat stress and wounding (3-fold). The presence of a large number of AP2 and bZIP TFBSs may play a significant role in regulating both biotic and abiotic stress responses.

The study conducted on *Oryza sativa* (rice) identified a total of 57 cis-acting regulatory elements (CAREs) in its promoter regions. These cis-elements varied in length from 4 to 10 base pairs (bp), with the majority being 6 to 7 bp long. The cis-elements were further categorized into different functional groups, revealing that a significant proportion (63%) were associated with stress responsiveness, with light-responsive elements constituting 34% of the stress-responsive group. The occurrence frequency of different cis-elements was nearly identical in both the forward and reverse strands of the 1-kilobase promoter region. Interestingly, the distribution analysis revealed that most cis-elements were clustered between 16 to 493 bp on the forward strand (+) and 546 to 842 bp on the reverse strand (−) (**Figure 5**).

Furthermore, specific cis-elements such as TATA, CAAT box (not shown in **Supplementary Table S3**), Box 4, and G-box were found to be more abundant in the *OsPR1* genes compared to other cis-elements. These findings provide valuable insights into the regulatory mechanisms governing the expression of *OsPR1* genes and highlight their responsiveness to various stress conditions in rice. The distribution of CAREs was unequal throughout the upstream region of the genes, with nearly equal numbers found in the proximal (<500 bp) and distal (>500 bp) regions. Gene expression is correlated with the presence of PCAREs and the number of transcription factor binding sites (TFBSs). Following SA, EBR, wounding, and heat stress treatment (HST), the *OsPR1-71* and *OsPR1-74* genes were upregulated and enriched with the greatest number of PCAREs. *OsPR1-74* was found to be enriched with defense-responsive TFBSs such as WRKY (32), AP2 (74), and developmentally related TFBSs such as TCP (186) and α-amylase (10) (**Supplementary Table 2**). *OsPR1-71* and *OsPR1-74* were enriched with W-box (TTGACC), SA-responsive element TCA (CCATCTTTTT), and EBR-binding element MBS (CAACTG), suggesting SA-mediated EBR expression of the genes (**Supplementary Table 3**). In addition, the *OsPR1-71, OsPR1-74, OsPR1-76*, and *OsPR1-21* genes, which were upregulated after 1 h and 4 h of heat stress treatment (HST), contain the highest number of hormone-responsive PCAREs. These genes were enriched with the maximum number of methyl jasmonate-responsive TGACG-motif and CGTCA-motif, suggesting their role in basal thermotolerance. Previous research in Arabidopsis has shown that jasmonic acid (JA) can mitigate the effects of high light and high temperature conditions, which is crucial for plants to thrive in various agroclimatic environments. Furthermore, *OsPR1-72* and *OsPR1-76* contain the GC-motif (CCCCCG), which is associated with anoxic-specific inducibility (**Supplementary Table 3**). This finding suggests that the *OsPR1* genes may be involved in submergence regulation.

**Figure 6:**
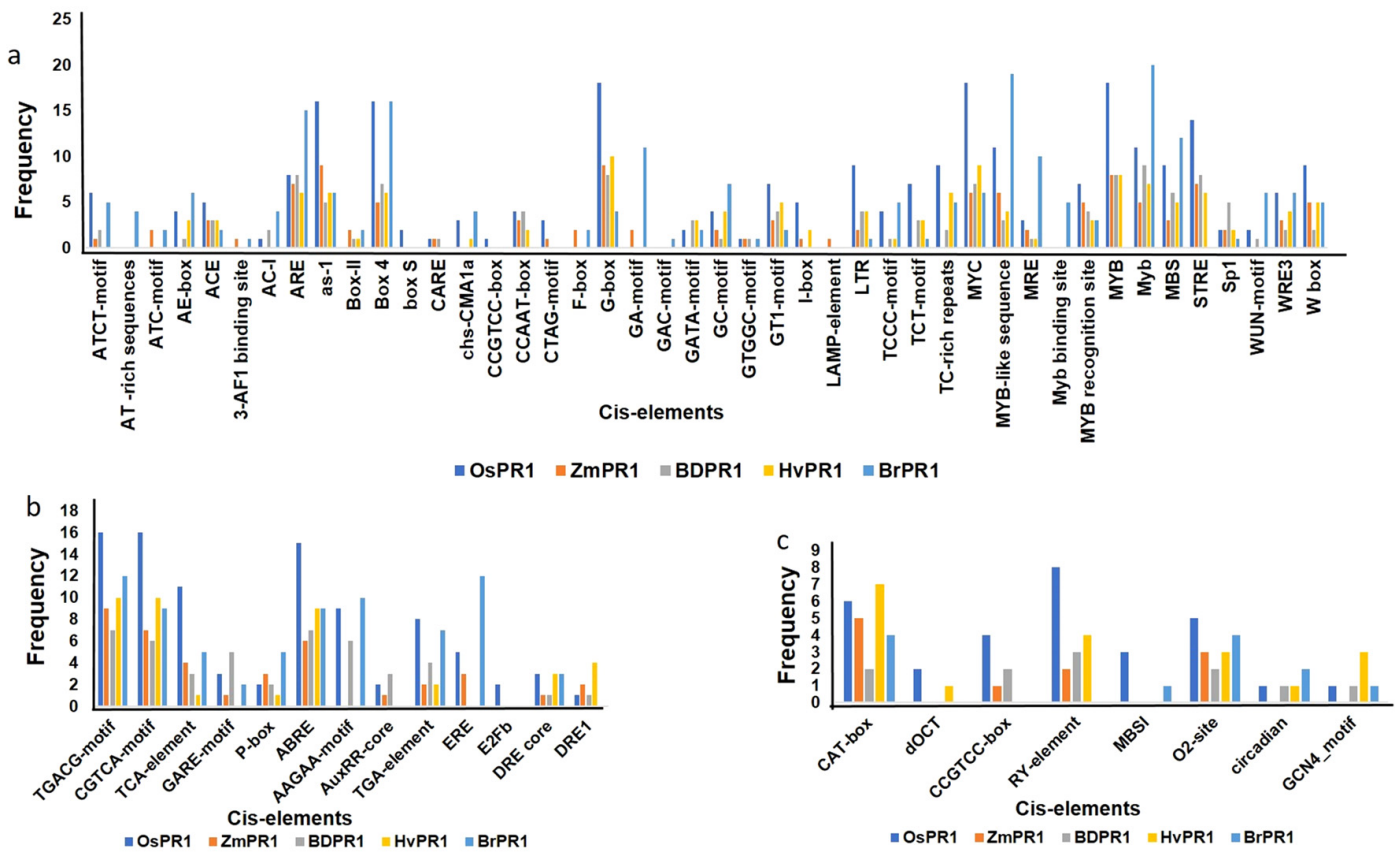
Frequencies of cis-regulatory motifs identified in the 1000 bp upstream promoter regions of Pathogenesis-Related 1 (PR1) genes in different plant species using PlantCARE. The figure presents the distribution of different categories of motifs identified in the promoter regions of PR1 genes in *Oryza sativa* (*OsPR1*), *Zea mays* (*ZmPR1*), *Brachypodium distachyon* (*BdPR1*), *Hordeum vulgare* (*HvPR1*), and *Brassica rapa* (*BrPR1*). **(A)** Stress-related motifs, such as W-box, TC-rich repeats, and LTR, are shown in the promoter regions across species. **(B)** Hormone-responsive motifs, including ABRE, TGA-element, and GARE-motif, indicating regulation by abscisic acid, salicylic acid, and gibberellins. **(C)** Tissue-specific motifs, such as CAT-box, RY-element, and O2-site, are compared across the PR1 genes of the different species, showing variability in their potential roles in developmental expression patterns.

**Figure 7:**
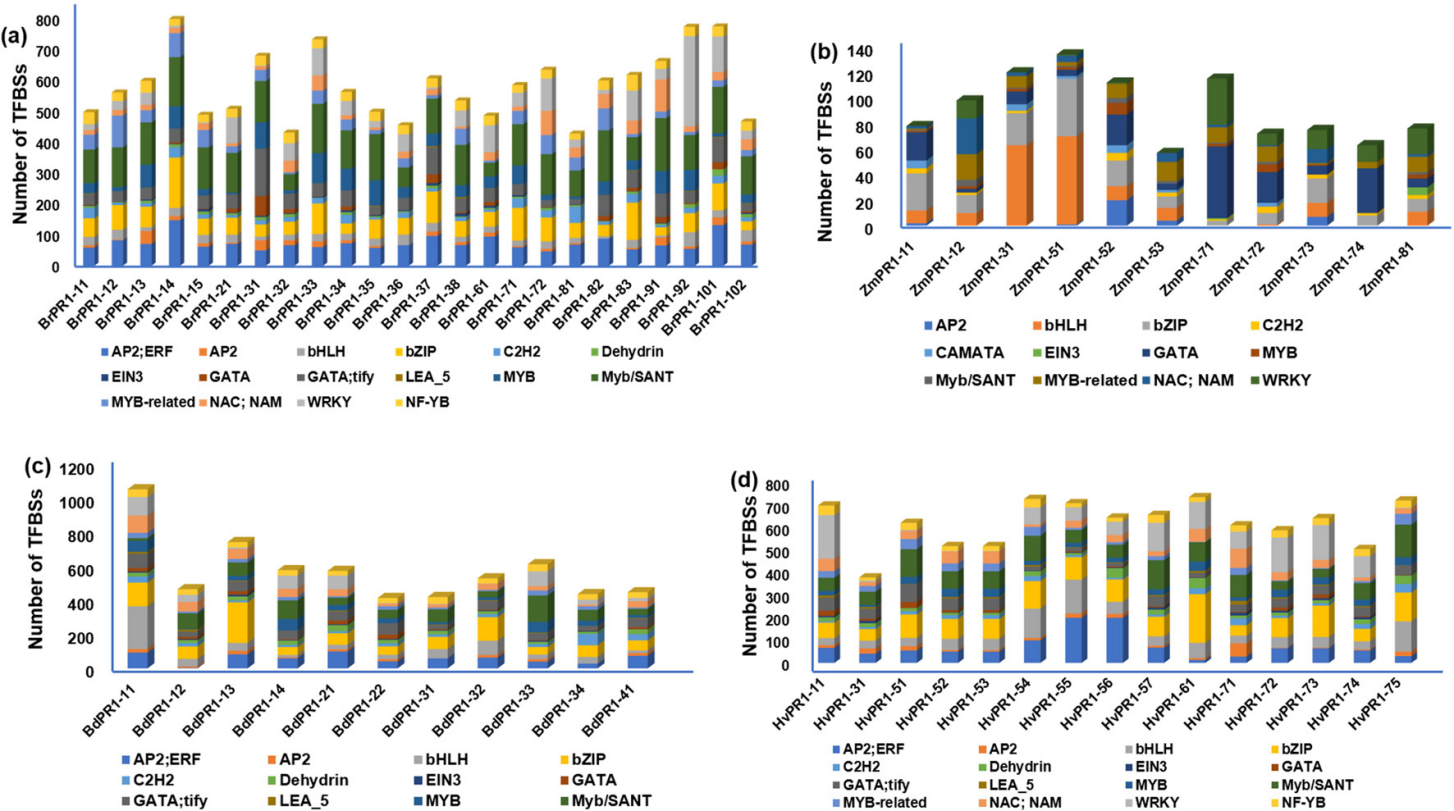
Distribution of transcription factor binding sites (TFBSs) in the 1000 bp upstream promoter regions of Pathogenesis-Related 1 (PR1) genes in different plant species. The graphs display the presence of various TFBS categories identified in PR1 gene promoter regions of **(A)** *Brassica rapa* (*BrPR1*), **(B)** *Zea mays* (*ZmPR1*), **(C)** *Brachypodium distachyon* (*BdPR1*), and **(D)** *Hordeum vulgare* (HvPR1). The color-coded bars represent the number of TFBSs associated with different transcription factor families, including *AP2/ERF, AP2, bHLH, bZIP, C2H2, EIN3, GATA, MYB, WRKY*, and others, indicating the complexity and variability of transcriptional regulation across different species.

The promoter sequences of *BdPR1, HvPR1, BrPR1*, and *ZmPR1* were studied to retrieve important stress-responsive TFBSs and CAREs. Comparatively, within the promoter sequences of all PR1 genes, stress-responsive TFBSs exhibit a higher prevalence compared to those associated with developmental processes. In *BdPR1*, *HvPR1*, and *BrPR1*, the number of total CAREs varies from 52 to 53, with 61% of them being stress-responsive. In contrast, *ZmPR1* has a total of 49 CAREs, with 70% being stress-responsive (**Supplementary Table 4**). The numbers of developmental-related CAREs in *BrPR1* and *ZmPR1* are compared to those in *BdPR1* and *HvPR1* (**Supplementary Table 4**, **Figure 6**). All the PR1 genes were enriched with TATA-box, CAAT-box, G-box, MYC, ARE, Myb, abscisic acid (CGTACGTGCA), and methyl-jasmonate (TGACG, CGTCA) responsive PCAREs (**Supplementary Table 4**). The maximum number of TFBSs was found in *BdPR1-11* [bHLH (252), bZIP (140), WRKY (109), NAC;NAM (102), BES1 (44), Myb-related (31), AP2;ERF (93)), followed by *BdPR1-13* (bZIP (239), EIN3 (11)] (**Figure 7**). In *ZmPR1*, the maximum enrichment of defense-responsive TFBSs was found in *ZmPR1-51* and *ZmPR1-31*, though the total enrichment was lower compared to others. In contrast, the enrichment of TFBSs in members of *HvPR1* and *BrPR1* was the highest (**Figure 7**).

## Discussion

Plant pathogens exhibit a remarkable range of ecological adaptations and can inflict substantial damage on plant growth, often surpassing the impact of many other organisms. While plants have not naturally evolved resistance to all diseases, researchers have implemented various strategies to enhance plant resistance. These approaches include modifying signaling pathways, gene pyramiding (stacking multiple resistance genes), and overexpressing disease-responsive genes, such as PR genes (Pathogenesis-Related genes) (Grover and Gowthaman, 2003). The studied PR1 genes belong to the CAP (Cysteine-rich Antifungal Protein) or SCP (Secretory Cysteine-rich Protein) family, which is known for its roles in the defense system, including ion binding, antifungal activity, cell wall degradation, sterol binding and transport, and reproduction (Schneiter and Di Pietro, 2013; Chen et al., 2014). The selection of these specific *PR1* genes was based on the presence of ten highly conserved amino acid residues in their C-terminal region. This conservation was observed not only in the *PR1* genes chosen for this study but also in all 36 PR1-type proteins identified across a diverse range of 14 plant species (Mitsuhara et al., 2008; Chen et al., 2014). PR1 proteins are thought to function in plants by utilizing regions in their C-terminus. In this study, out of the 11 *OsPR1* genes selected for expression analysis, 9 exhibited upregulation following SA treatment. In numerous studies, *PR1* has been shown to play a significant role in plant defense by acting as a signaling marker for SA pathways. Under optimal environmental conditions, healthy and young rice leaves contain high amounts of salicylic acid (SA), with levels reaching up to 10 µg per gram of fresh leaf. In contrast, tobacco and Arabidopsis leaves contain much lower amounts of SA, approximately 20 ng and 30 ng, respectively (Silverman et al., 1995; Park et al., 2007; Seo et al., 2007). Rice leaves are known to possess remarkably high levels of SA, ranging from 50 to 500 times greater than those found in tobacco or Arabidopsis leaves. These SA levels in rice are comparable to the levels induced by Tobacco mosaic virus (TMV) infection, which typically leads to hypersensitive response (HR) lesion formation. Given the elevated SA levels in rice, it is conceivable that the expression of *OsPR1* genes could be significantly influenced by treatments with SA, EBR, heat stress (HS), and wounding (Mitsuhara et al., 2008). To compare the nature of 11 *OsPR1* genes, the results are summarized in **Supplementary Table 1**. The phylogenetic analysis reveals that *OsPR1*#*074 (OsPR1a)* and *OsPR1-74* fall into the same clade, exhibiting similar characteristics and forming transcripts after all treatments. *OsPR1-74* could act as a common marker for multiple stresses. *OsPR1*#*011 (OsPR1b)* was found to be upregulated after SA treatment but downregulated after wounding. Similar trends were observed in *OsPR1-75*, which is located close to *OsPR #011* in the phylogenetic tree. The process of wounding is known to activate numerous genes, including *PR1* genes, as demonstrated in studies by Mitsuhara et al. (2008) and Ali et al. (2018b). Investigating the expression pattern of *OsPR1* genes under wounding treatment in rice holds a significant promise in uncovering the detailed role of these genes under various stress conditions. Investigating the expression pattern of *OsPR1* genes under wounding treatment in rice holds significant promise for uncovering the detailed roles of these genes under various stress conditions. *OsPR1* genes are typically induced by biotic stresses, such as pathogen infection. Upon infection with *X. oryzae* pv. *oryzae* (*Xoo*), *OsPR1* expression is significantly upregulated, which correlates with an enhanced defense response in rice plants (Mitsuhara et al., 2008). Among these PR1 genes, *OsPR1a* is a well-characterized member known to be involved in rice defense mechanisms (Yan et al., 2022). Investigating the expression of other *OsPR1* genes were upregulated after treatment.

By understanding how *OsPR1* responds to wounding stress, we can gain valuable insights into its broader functions during different types of stress in rice. In the present study, 5 *OsPR1* genes were upregulated after wounding, 24 hours post-treatment, indicating a late wounding response. Both early and late inducible wounding-responsive *PR1* genes have been reported in tomato and *B. napus* (Scranton et al., 2013; Ali et al., 2018). Additionally, it has been reported that wounding and the defense response utilize similar signaling pathways, including SA, JA, and ET (Maleck, 1999). The expression profile of *OsPR1* genes was found to be similar to that observed in Mitsuhara et al. (2008) study.

Brassinosteroids (BRs) mediated response in multiple stress is extensively studied but PR1 mediated response need to be verified in rice (Li and Chory, 1999; Sreeramulu et al., 2013). Previous studies, conducted by Nakashita et al. (2003), Divi et al. (2010), and Sahni et al. (2016), have demonstrated that the application of exogenous brassinosteroids (BR) enhances overall plant resistance against pathogens. These findings highlight the positive impact of BR treatment on bolstering plant defenses against various pathogens. Similarly, we have studied the *OsPR1* response after exogenous treatment of BR and found that six genes along with *OsPR1a* upregulated after treatment. Further, we found that 4 *OsPR1* genes (*OsPR1*-22, *OsPR1*-61, *OsPR1*-74, *OsPR1*-77) were upregulated after all the treatments. All the *PR1* genes that were upregulated in heat stress follow the same trend in BR treatment. However, the observed downregulation of *OsPR1-76* after BR treatment might suggest that *OsPR1-76* is not a BR-responsive gene. In brassica, several overlapping findings came in light of how BR modulated the expression of WRKY, PR, HSP, protein synthesis, calcium signaling, and ROS-associated genes (Sahni et al., 2016) as well as multiple stress responsive genes. In Arabidopsis *cpd* mutants, which are deficient in BR biosynthesis, a decrease in the *PR1, PR2*, and *PR5*, whereas the CPD over-expressed transgenic line shows higher expression (Szekeres et al., 1996), suggesting that BL induces *PR* gene expression. Contrary to its effects in rice and other plants, exogenous application of brassinosteroids (BR) did not result in changes in the expression of *PR* genes in tobacco, as evidenced by the study conducted by Nakashita et al. (2003). This observation suggests that *NPR1*, a key regulator of SA-mediated defense genes, may play a crucial role in the enhancement of thermotolerance and salt tolerance triggered by BR. However, *NPR1* is not necessary for the BR-mediated induction of *PR-1* gene expression in tobacco. This finding indicates that BR can exert its anti-stress effects independently, and it may also interact with other hormonal pathways to elicit its effects, as reported by Divi et al. (2010). Thus, BR appears to modulate stress responses through diverse mechanisms, and its interactions with other hormones contribute to its multifaceted effects on plant defense and stress tolerance. In this experiment, *OsPR1-78* gene showed enhanced expression (~12.8-fold) after EBR treatment, whereas no change in expression was observed after SA, HS, or wounding treatment. So, in future it is need to study critically the link between BR and SA pathway.

Moreover, *PR1* gene has role in multiple type of abiotic stress, but PR1 gene expression after abiotic stress has not been studied in rice. For the first time, we studied the 11 *OsPR1* gene expression after heat stress treatment. In this study, we found that four *OsPR1* genes along with *OsPR1a* gene were upregulated after 1 h and 4 h of heat stress treatment, whereas 6 and 7 *OsPR1* individually upregulated in 1 h and 4 h of heat stress treatment, respectively. *OsPR1-76* expressed maximally after 4 h of HS treatment indicates that *OsPR1* have a role in amelioration effect on plants against heat stress. It is also believed that high temperature is responsible for rice’s susceptibility to pathogens (Dossa et al., 2017). *PR1* gene expression in abiotic stressed condition studied in many crops like wheat, Arabidopsis, tomato, grape (Liu et al., 2013; Wang et al., 2019; Akbudak et al., 2020; Li et al., 2021) help to study the regulation of gene. *TaPR-1-1* gene expression studied in wheat after osmotic stress, freezing, and salinity treatment and overexpression of *TaPR-1-1* leads to stress tolerance response in Arabidopsis and yeast (Wang et al., 2019). In Arabidopsis, the *Di19* (drought-induced) protein has been shown to positively regulate the expression of pathogenesis-related genes, specifically *PR-1, PR-2*, and *PR-5* (Liu et al., 2013). 13 *SlPR-1* reported in tomato, each gene leads to upregulation (as high as 50-fold) after drought stress treatment (Akbudak et al., 2020). Additionally, the genes *PR1a2* and *PR1b1* in tomato were consistently upregulated after 12- and 24-hour heat stress treatments, and heat shock element motifs were detected in their promoters (Arofatullah et al., 2018). In grape, the expression of *VvHSP24*, a class B heat shock protein has been found to be regulated by *PR1*. In transgenic Arabidopsis thaliana plants expressing *VvNPR1* (*NPR1* from *Vitis vinifera*) along with several salicylic acid (SA)-inducible genes, including *PR1, PR2*, and *PR5*, a notable decrease in resistance to *Botrytis cinerea* (a fungal pathogen) was observed compared to wildtype plants. The overexpression of *VvNPR1* and these SA-inducible genes seemingly compromised the plant’s defense response, leading to reduced resistance against *B. cinerea* infection when compared to the natural or wildtype counterparts (Li et al., 2021). It has been reported that *HuPR-1* is up-regulated by heat stress treatment, and it can also be induced by salt stress (Nong et al., 2019) and overexpressing of *HuPR-1* in Arabidopsis results in tolerance to heat and salt stress. Pepper PR-1 protein shows both biotic and abiotic tolerance (Sarowar et al., 2005). These results provide new insights into heat stress regulation that could lead to a better understanding of how *PR-1* genes are regulated in response to abiotic stresses in future studies (Jiao et al., 2021). These studies indicated that *PR* genes function not only in responses to biotic stress, but also in response to abiotic stress in rice.

### Role of TFBSs in stress response

The specific binding of transcription factors (TFs) to their respective transcription factor binding sites (TFBSs) is a crucial mechanism for mediating the transcriptional regulation of target genes. This process allows TFs to exert precise control over gene expression by interacting with specific DNA sequences in the regulatory regions of their target genes (Narlikar and Ovcharenko, 2009; Jayaram et al., 2016). The evidence of high throughput experimental methods accelerate the identification of TFBSs (Jayaram et al., 2016). However, in silico identification of TFBSs was still a method of choice to study various defense makers of plants (Boeva, 2016; Kumari et al., 2018). *PR*1 genes were acting as defense markers through activating a series of elements including transcription factors, MAPK and ROS. PR1 is a family of proteins whose expression regulates different functions during stress. The in silico analysis of the promoter regions of *PR1* genes revealed the presence of multiple cis-regulatory elements, providing strong evidence for their involvement in multiple stress responses.

WRKY (W box) has an extensive role in biotic and abiotic stress along with a wide range of development process (Choi et al., 2015; Rinerson et al., 2015). Different *PR1* genes of this study enriched with conserved consensus sequence TTGAC(C/T/A/G) (**Supplementary Table 3**). WRKY proteins play a crucial and diverse role in plant responses to various stresses and are involved in essential processes related to plant development, embryogenesis, dormancy, as well as responses to abiotic stresses like drought, salt, and heat in rice (Kalde et al., 2003; Jiang and Deyholos, 2009; Chen et al., 2012; Rushton et al., 2012). In addition, *OsWRKY03* regulates a cascade of Jasmonic acid (JA) and salicylic acid signaling pathways to protect plants against bacterial and fungal infections (Liu et al., 2005). WRKY have been shown to exert a positive regulatory effect on the heat tolerance of plants. For example, in pepper plants, *CaWRKY40* plays a vital role in the response to high-temperature stresses, contributing to enhanced heat tolerance. Similarly, in rice, *OsWRKY11* is involved in the plant’s resistance to heat stress, further emphasizing the significance of WRKY in facilitating heat stress adaptation in different plant species (Wu et al., 2009; Cai et al., 2015). In Arabidopsis, it was observed that *PR1* genes expression levels were notably higher in plants overexpressing *WRKY39* compared to wild-type plants following heat treatment. This finding led researchers to speculate that *WRKY39* likely acts as an upstream regulator of *PR1*, influencing its transcription and subsequently contributing to the plant’s response to heat stress (Li et al., 2010).

The basic region/leucine zipper motif (bZIP) plays a crucial role in regulating diverse stress and developmental responses in plants. These bZIP proteins exert their regulatory functions by binding to specific transcription factor binding sites (TFBSs) on DNA. Notably, plant bZIP proteins have a preference for recognizing the ACGT core in DNA sequences, particularly in regions known as the A-box, C-box, and G-box (Jakoby et al., 2002), CCAAT-box, TGA-element, NON, ABRE (Holdsworth et al., 1995; Jakoby et al., 2002; Liao et al., 2008) AS-1 (Després et al., 2000), TATCCAT/C-motif (Kong et al., 2018). G-box element imparts response to abscisic acid ABRE (ABA-responsive element), methyl-jasmonate, Anaerobiosis, defense responses ethylene induction as well as in seed specific expression found in most of studied *PR1* genes (Kong et al., 2018). The TATCCAT/C-motif (Kong et al., 2018) and TGACGTCA, commonly referred to as the G-box, is abundantly present in the promoter regions of many stress-inducible genes, including the *PR1* promoter. This interplay between SA, the TGA subfamily, and *NPR1* represents a crucial mechanism underlying the activation of promoters involved in the plant’s defense against pathogens (Zander et al., 2014). In a previous report were found that AS-1 cis element is an oxidative stress-responsive element and activated by SA by binding TGA cis element (Zander et al., 2014). bZIP proteins have impart their major role in activating a number of defense genes (Alves et al., 2013).

AP2 is one of the biggest plant TF super families which play a significant role in regulating plant growth and stress responses (Najafi et al., 2018). The AP2 (APETALA2)/ERF family is categorized into three distinct subfamilies based on the number of AP2/ERF domains they possess. These subfamilies are known as AP2, ethylene responsive factor (ERF), and RAV. Each subfamily is distinguished by its unique arrangement of AP2/ERF domains (Nakano et al., 2014). The ERF subfamily plays a crucial role in regulating a wide array of genes associated with both biotic and abiotic stresses in rice. These stresses include challenging environmental conditions like drought, high salinity, and cold temperatures (Pandey et al., 2014). The AP2 subfamily is known to be involved in regulations of developmental process such as flower development, ovule development, seed set and regulating organ-specific growth while RAV subfamily genes response to expression induced by ethylene, brassinosteroids, and biotic and abiotic stresses (Xu et al., 2013). ERF subfamily members bind to the conserved nucleotide sequences AGCCGCC (Xu et al., 2013) and TACCGACAT (GCC-box) (Pandey et al., 2014) in the upstream regions of genes while the RAV binds to CAACA and CACCTG motif (Song et al., 2013). AP2/ERF transcription factors have been identified as regulators that can trigger the synthesis of ethylene (ET), salicylic acid (SA), and jasmonic acid (JA). These signaling molecules play a significant role in enhancing the expression of pathogenesis-related (*PR*) genes during instances of pathogen infestation (Singh, 2002; Brown et al., 2003). Moreover, the presence of AP2/ERF transcription factor binding sites (TFBSs) in defense-responsive genes has been found to strengthen resistance against specific biotic and abiotic stresses (Berrocal-Lobo et al., 2002). The ERF family of TFs was shown to regulate abiotic and biotic stresses (Pandey et al., 2014) as well as involved in ethylene mediated *PR* genes expression which confer tolerance against cold and dehydration stresses (Liu et al., 2007). In the AP2/EREBP family, a subset called DREBs is classified under the EREBP subfamily and plays a pivotal role in plants’ response to various abiotic stresses. Notably, specific DREB members, such as rice *OsDREB2B* and maize *ZmDREB2A*, are known to be induced and expressed in response to high-temperature stress. This induction of DREBs assists plants in coping with the challenges posed by elevated temperature conditions (Qin et al., 2007; Matsukura et al., 2010). CG-1;CAMTAs is comprised of core sequence CGTG (Silva, 1994) which associated with ABRE cis- element. In our study of PR1 genes we found the same consensus sequence and another cis-element chs-CMA1a (TTACTTAA) were also present. Calmodulin binding transcription activators (CAMTAs) are proteins that exhibit responsiveness to a diverse range of external signals, including cold, wounding, and drought. Additionally, they are influenced by hormonal signals like ethylene and ABA (Reddy et al., 2000; Yang and Poovaiah, 2003). Our research findings indicate that Calmodulin signaling responsive genes are predominantly linked to ABRE (CGTG) cis-elements.

This may be possible that *PR1* signaling response is linked to ABA hormone signaling. bHLH can recognize the MYC (CATTTG), G-box, E-box, WRE3 (CACCT) (Miyamoto et al., 2012). bHLH transcription factors (TFs) play a crucial role in various aspects of plant biology, including SA and JA mediated stress responses, light-induced hormone signaling, wound and drought stress management, shoot branching, as well as fruit and flower development, root development, and other pathways (Carretero-Paulet et al., 2010). Moreover, bHLH TFs are also involved in inducing ABA-dependent signaling to cope with cold stress, specifically in rice (Chinnusamy et al., 2003). MYB transcription factors (TFs) are universally present in eukaryotes and are associated with specific TFBS sequences, such as TAACTG, CAACTG, AACGG, and C/TAACNA/G, as reported by Xu et al. (2008) and Qin et al. (2012). These MYBs play essential roles in various biological processes, including plant development, metabolism, and stress responses. In rice, MYB TFs have been demonstrated to be crucial in managing abiotic stresses like dehydration, salt, and cold stresses (Abe et al., 1997). They are also involved in the ABA and SA-signaling pathways, which confer responses to both biotic and abiotic stresses (Ambawat et al., 2013). One specific member of the MYB family, *OsMYB55*, has been shown to enhance the tolerance of rice plants to high temperatures. This is achieved by increasing the expression of downstream genes like *OsGS1;2, GAT1*, and *GAD3*, which are involved in amino acid metabolism (El-Kereamy et al., 2015).

TCP proteins possess a distinctive TCP domain, and their binding sites exhibit variation, including sequences like GGNCCCAC, GTGGNCCC, and TGGGCC, as identified by Giraud et al. (2011). These TCP genes play a crucial role in influencing the development of axillary structures, petals, and stamens in plants (Cubas et al., 1999). The SBP protein has been shown to interact with the GTAC core sequence, as demonstrated by Wang et al. (2015). In plants, the SBP protein is actively involved in various developmental processes, including flower and fruit development, architecture formation, responses to copper and fungal toxins, as well as regulation of GA (gibberellic acid) levels (Wang et al., 2009). B3 cis -elements are combining with core sequences of CATGC (RY-element) verified in all *PR1* genes. The B3 family of proteins plays significant roles in regulating flowering time, organ growth, and organ polarity in plants. These B3 members are distributed in various plant tissues, including pistils, stamens, germinated seeds, and roots (Swaminathan et al., 2008). Together with these TFBSs, many more found in *PR1* genes, which regulate the multiple stress along with developmental process.

## Conclusion

This study provides valuable insights into the multifaceted nature of PR1 proteins. While these proteins play a crucial role in connecting biotic and abiotic stress responses, certain aspects of their characteristics remain unexplored and require further investigation. There is a requirement for further expansions in the classification of PR-proteins, extending the existing 17 families. The considerable expansion of the PR proteins and the differential expression of several *OsPR1* genes in response to abiotic and biotic stresses supports the importance of further investigating *PR1* genes as targets for increasing stress tolerance in crop plants. Several PR1 proteins, which may play a role in plant defense mechanisms, have been uncovered from our study. We detected PR1 homologues in different crops by performing computational prediction analysis and querying the PR1 protein dataset from *OsPR1*. These 58 putative PR1 proteins shared sequence, subcellular localization, SCP domain, similarities with the query PR1 proteins from *OsPR1*. The expression characteristic studied in eleven *OsPR1* genes after SA, wounding, BR, HS treatment, which proves its predominant role as a marker of biotic and abiotic stress. The expression profiling reveals the evident role in the SA-dependent pathway. Additionally, critical promoter analysis of 1000 bp was performed to find the role in TFBSs in response to multiple stress. In short, the findings of this study may provide new insight into the regulatory mechanism of *OsPR1* in response to BR and HS that could aid future study to examine the role of *OsPR1* genes in response to different abiotic stress. The *in-silico* promoter analysis of *O*. *sativa, Z. mays, B. distachyon, H. vulgare, B. rapa* further can provide plant genetic engineering technology for protection of crops against biotic and abiotic stress.

## Data Availability

The original contributions presented in the study are included in the article/**Supplementary Material**, further inquiries can be directed to the corresponding authors.

## Supplementary material

Supplementary Figure 1 Workflow of promoter analysis of PR1 genes in different crops. A complete analysis of promoter region involves various steps which start from downloading naïve protein sequencing. The major steps shown in screenshot of regions which link the steps. The users may change the species name by selecting species name in Phytozome12. Tool. Downloaded protein sequence cross was verified in Phytozome12. Tool afterwards promoter sequence was retrieved from genomic region.

Supplementary Table 1 List of OsPR1 Primers used in gene expression analysis.

Supplementary Table 2 Characterization of Transcription Factor Binding Sites in Promoter Regions of Rice Pathogenesis-Related (OsPR1) Genes.

Supplementary Table 3 Identification and Functional Categorization of Cis-Elements in the 1000 bp Upstream Promoter Region of Pathogenesis-Related 1 (PR1) Genes in (*Oryza sativa*).

Supplementary Table 4 Comparative Analysis and Categorization of Cis-Elements in the 1000 bp Upstream Promoter Regions of Pathogenesis-Related 1 (PR1) Genes Across *Zea mays*, Brachypodium distachyon, Hordeum vulgare, and Brassica rapa.

Supplementary Table 5 List of Genes taken for Search BLASTP search of other genes.

Supplementary Table 6 List of Genes obtained after BLASTP search.

Supplementary Table 7 List of Genes obtained after HMM search.
