# Supplementary material for: Comprehensive analysis of transcription factor binding sites and expression profiling of rice pathogenesis related genes (OsPR1)
Source: Front Plant Sci. 2024 Oct 25;15:1463147. doi: 10.3389/fpls.2024.1463147 (PMC11543534; doi:10.3389/fpls.2024.1463147)
Supplement: Supplementary Figure 1 — Workflow of promoter analysis of PR1 genes in different crops. A complete analysis of promoter region involves various steps which start from downloading naïve protein sequencing. The major steps shown in screenshot of regions which link the steps. The users may change the species name by selecting species name in Phytozome12. Tool. Downloaded protein sequence cross was verified in Phytozome12. Tool afterwards promoter sequence was retrieved from genomic region. [file DataSheet1.docx]

**
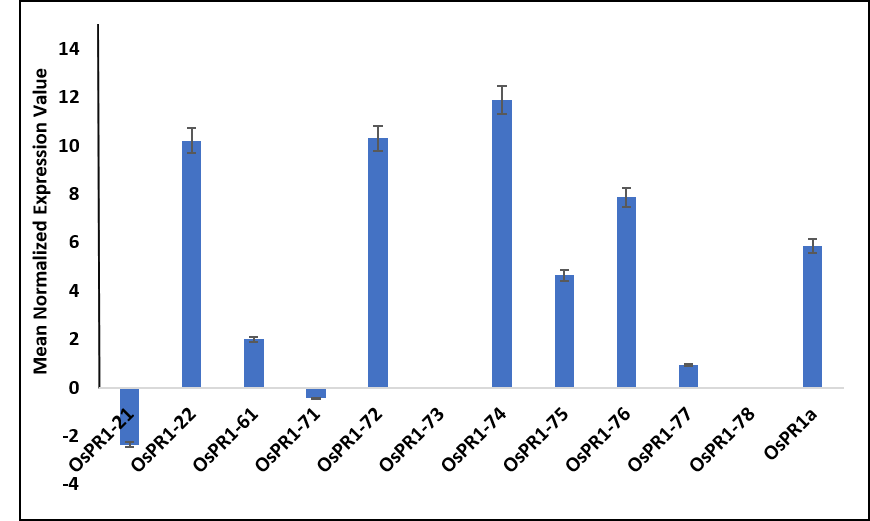
**

**Supplementary Fig. 1.** Rice seedlings were infected with X. oryzae pv. oryzae, and the transcript levels of *OsPR1* genes were analyzed using qRT-PCR at 24h post-infection. Relative transcript abundance is presented as fold-change compared to the mock treatment, with *OsPR1a* expression used as a positive control. Results are representative of three independent experiments, with error bars indicating the standard error of the mean for three replicates.

**Supplementary Table 1: List of OsPR1 Primers used in gene expression analysis**

| **Protein ID/ Proposed ID** | **Forward primer (5’-3’)** | **Reverse primer (5’-3’)** |
| --- | --- | --- |
| LOC_Os02g54530.1 (OsPR1-21) | GTGATCCTCCTCCTCCAC | GAACAGGTTCTCCCCGTA |
| LOC_Os02g54570.1 (OsPR1-22) | ACCAGTACAAGGGCGACT | GCCACATCATCTGCTTGT |
| LOC_Os06g24290.1 (OsPR1-61) | CCGTGCTGCAATAGTAATCT | GTAGATGTTCTCCCCATACG |
| LOC_Os07g03409.1 (OsPR1-71) | CTTGTGGATGGAGGAGAAG | TAGTTGCAGCTGATGATGAC |
| LOC_Os07g03600.1 (OsPR1-72) | CAAATGGATGGAGGAGAAG | TAGTTGCAGCTGATGATGAC |
| LOC_Os07g03680.1 (OsPR1-73) | TACGGGGAGAACATCTACG | AGTTGCAGATGACGAAGAC |
| LOC_Os07g03690.1 (OsPR1-74) | AAGCAGTGGTACAACCACAC | GTAGTTGCAGGTGATGAACA |
| LOC_Os07g03730.1 (OsPR1-75) | AAGTACGGCGAGAACATCT | CGAGTAGTTGCAGGTGATG |
| LOC_Os07g03740.1 (OsPR1-76) | CAAGGTGGTGTTAAGAAAGG | ATACATCCGTCCACAAGAAG |
| LOC_Os07g14030.1 (OsPR1-77) | GTCAAGCTCCACAACGAC | CGATCGTAGTACGGCTTCT |
| LOC_Os07g14070.1 (OsPR1-78) | GAAGTGTACCGAAGAAAGGA | CCCTCTTGTTTCAATGCTAA |
| OsPR1a | ATGGCACCTTCCAAGGTCAG | TGCAGGTTGACGTAGTCCTG |
| OsActin | GAGTATGATGAGTCGGGTCCAG | ACACCAACAATCCCAAACAGAG |

**Supplementary Table 2: Description of TFBSs in Rice Pathogenesis related (*OsPR*1) genes in promoter regions.**

| GENE | AP2 | bHLH | bZIP | C2H2 | EIN3 | GATA | LEA_5 | MYB | Myb/SANT | NAC; NAM | WRKY | NF-YB | AT-Hook | MADF | MADS box; MIKC; M-type | SBP | TBP | TCP | TCR | Alpha-amylase | B3 |
| --- | --- | --- | --- | --- | --- | --- | --- | --- | --- | --- | --- | --- | --- | --- | --- | --- | --- | --- | --- | --- | --- |
| OsPR1#011 | 1 | 2 | 4 | 7 | 5 | 5 | 4 | 2 | 53 | 1 | 0 | 38 | 20 | 2 | 21 | 32 | 0 | 47 | 1 | 10 | 22 |
| OsPR1# 012 | 0 | 6 | 26 | 0 | 13 | 6 | 7 | 2 | 11 | 1 | 1 | 34 | 13 | 2 | 2 | 20 | 15 | 53 | 0 | 10 | 45 |
| OsPR1# 021 | 4 | 6 | 6 | 0 | 1 | 6 | 0 | 3 | 16 | 4 | 8 | 34 | 14 | 2 | 1 | 6 | 2 | 56 | 0 | 10 | 29 |
| OsPR1# 022 | 1 | 15 | 2 | 5 | 0 | 0 | 4 | 1 | 29 | 4 | 6 | 28 | 34 | 5 | 0 | 44 | 0 | 39 | 4 | 18 | 35 |
| OsPR1# 051 | 12 | 8 | 5 | 6 | 6 | 19 | 2 | 0 | 6 | 6 | 0 | 0 | 6 | 1 | 0 | 26 | 11 | 71 | 0 | 3 | 38 |
| OsPR1# 052 | 7 | 7 | 19 | 2 | 2 | 13 | 3 | 1 | 21 | 2 | 0 | 26 | 10 | 0 | 0 | 20 | 8 | 63 | 0 | 10 | 21 |
| OsPR1# 071 | 3 | 15 | 6 | 2 | 7 | 1 | 6 | 2 | 13 | 11 | 9 | 41 | 30 | 0 | 1 | 4 | 15 | 56 | 2 | 10 | 40 |
| OsPR1# 072 | 5 | 74 | 41 | 6 | 2 | 4 | 4 | 2 | 18 | 6 | 34 | 32 | 19 | 1 | 1 | 79 | 28 | 38 | 3 | 13 | 24 |
| OsPR1# 073 | 5 | 6 | 5 | 4 | 0 | 18 | 2 | 3 | 21 | 0 | 0 | 37 | 10 | 0 | 0 | 4 | 9 | 74 | 2 | 14 | 28 |
| OsPR1# 074 | 4 | 5 | 23 | 2 | 5 | 9 | 4 | 1 | 10 | 10 | 0 | 35 | 28 | 0 | 0 | 62 | 41 | 34 | 34 | 18 | 35 |
| OsPR1# 101 | 11 | 1 | 4 | 11 | 2 | 1 | 3 | 1 | 21 | 1 | 7 | 31 | 5 | 1 | 0 | 3 | 15 | 49 | 3 | 7 | 32 |
| OsPR1# 121 | 1 | 2 | 4 | 7 | 5 | 5 | 4 | 2 | 53 | 1 | 1 | 38 | 20 | 2 | 0 | 33 | 1 | 47 | 1 | 9 | 22 |
| OsPR1-21 | 0 | 12 | 8 | 0 | 5 | 19 | 1 | 2 | 5 | 5 | 0 | 37 | 14 | 1 | 1 | 20 | 10 | 81 | 0 | 3 | 39 |
| OsPR1-22 | 21 | 11 | 11 | 8 | 0 | 41 | 7 | 2 | 4 | 2 | 17 | 34 | 7 | 1 | 3 | 16 | 5 | 45 | 0 | 6 | 47 |
| OsPR1-61 | 7 | 2 | 9 | 6 | 0 | 33 | 1 | 4 | 28 | 3 | 9 | 30 | 9 | 0 | 0 | 1 | 11 | 89 | 0 | 8 | 39 |
| OsPR1-71 | 0 | 5 | 26 | 14 | 8 | 6 | 5 | 2 | 9 | 5 | 11 | 26 | 10 | 2 | 2 | 14 | 14 | 44 | 1 | 9 | 34 |
| OsPR1-72 | 5 | 63 | 38 | 5 | 0 | 10 | 3 | 1 | 7 | 6 | 88 | 30 | 22 | 1 | 1 | 24 | 9 | 65 | 1 | 5 | 34 |
| OsPR1-73 | 1 | 3 | 4 | 5 | 3 | 6 | 1 | 3 | 13 | 7 | 93 | 32 | 18 | 7 | 1 | 22 | 28 | 39 | 0 | 16 | 18 |
| OsPR1-74 | 31 | 6 | 12 | 4 | 10 | 28 | 6 | 0 | 10 | 3 | 0 | 33 | 16 | 2 | 6 | 4 | 13 | 61 | 0 | 10 | 45 |
| OsPR1-75 | 19 | 15 | 29 | 0 | 5 | 33 | 7 | 2 | 18 | 5 | 0 | 27 | 16 | 0 | 0 | 45 | 7 | 33 | 0 | 10 | 48 |
| OsPR1-76 | 175 | 71 | 61 | 1 | 0 | 0 | 0 | 0 | 8 | 0 | 26 | 23 | 0 | 0 | 0 | 0 | 2 | 186 | 0 | 0 | 27 |
| OsPR1-77 | 3 | 4 | 33 | 2 | 4 | 8 | 4 | 3 | 11 | 0 | 10 | 26 | 6 | 1 | 3 | 2 | 14 | 41 | 0 | 5 | 50 |
| OsPR1-78 | 5 | 1 | 21 | 5 | 3 | 6 | 0 | 0 | 5 | 3 | 0 | 26 | 16 | 1 | 1 | 3 | 7 | 117 | 1 | 0 | 40 |

**Supplementary Table 3: Category wise list of cis-elements extracted from 1000 bp upstream region of *OsPR1* genes using Plant Care**

| **STRESS** |  |  |  |
| --- | --- | --- | --- |
| **Motifs** | **Consequence** | **Function** | **Accession** |
| ATCT-motif | AATCTAATCC | light responsiveness | OsPR1#011, OsPR1#021, OsPR1#074, OsPR1#121, OsPR1-75, OsPR1-74 |
| GT1-motif | GGTTAAT | light responsive element | OsPR1#011, OsPR1#021, OsPR1#022, OsPR1#051, OsPR1-21, OsPR1-22, OsPR1-73 |
| MYC | CATTTG |  | OsPR1#011, OsPR1#012, OsPR1#021, OsPR1#022, OsPR1#051, OsPR1#071, OsPR1#072, OsPR1#073, OsPR1#074, OsPR1#101, OsPR1#121, OsPR1-21, OsPR1-22, OsPR1-78, OsPR1-72, OsPR1-75, OsPR1-71, OsPR1-74 |
| TCCC-motif | TCTCCCT | light responsive element | OsPR1#052, OsPR1#101, OsPR1#121, OsPR1-76 |
| TCT-motif | TCTTAC | light responsive element | OsPR1#011, OsPR1#051, OsPR1#052, OsPR1#073, OsPR1#121, OsPR1-77, OsPR1-78 |
| Box 4 | ATTAAT | part of a conserved DNA module involved in light responsiveness | OsPR1#011, OsPR1#022, OsPR1#051, OsPR1#052, OsPR1#071, OsPR1#072, OsPR1-74, OsPR1#073, OsPR1#074, OsPR1#101, OsPR1-22, OsPR1-61, OsPR1-73, OsPR1-78, OsPR1-72, OsPR1-71 |
| G-box | TACGTG | cis-acting regulatory element involved in light responsiveness | OsPR1#011, OsPR1#012, OsPR1#021, OsPR1#022, OsPR1#051, OsPR1#052, OsPR1#071, OsPR1#072, OsPR1#074, OsPR1#101, OsPR1#121, OsPR1-21, OsPR1-77, OsPR1-22, OsPR1-78, OsPR1-72, OsPR1-75, OsPR1-71 |
| GATA-motif | GATAGGG | light responsive element | OsPR1#101, OsPR1-78 |
| AE-box | AGAAACAA | light response | OsPR1#012, OsPR1#071, OsPR1-61, OsPR1-78 |
| ACE | GACACGTATG | cis-acting element involved in light responsiveness | OsPR1#011, OsPR1#074, OsPR1#052, OsPR1#101, OsPR1-76 |
| MRE | AACCTAA | MYB binding site involved in light responsiveness | OsPR1#052, OsPR1#022, OsPR1-78 |
| I-box | cGATAAGGCG | light responsive element | OsPR1#071, OsPR1#073, OsPR1#101, OsPR1-71, OsPR1-74 |
| ARE | AAACCA | the anaerobic induction | OsPR1#121, OsPR1#022, OsPR1#071, OsPR1-77, OsPR1-73, OsPR1-78, OsPR1-71, OsPR1-74 |
| box S | AGCCACC |  | OsPR1#022, OsPR1-77 |
| WRE3 | CCACCT |  | OsPR1#071, OsPR1#011, OsPR1#052, OsPR1-21, OsPR1-76, OsPR1-72 |
| W box | TTGACC |  | OsPR1#021, OsPR1#071, OsPR1#072, OsPR1#101, OsPR1-76, OsPR1-61, OsPR1-73, OsPR1-72, OsPR1-71 |
| CARE | CAACTCCC |  | OsPR1#021 |
| MYB-like sequence | TAACCA |  | OsPR1#051, OsPR1#121, OsPR1#052, OsPR1#022, OsPR1#071, OsPR1-77, OsPR1-22, OsPR1-61, OsPR1-73, OsPR1-78, OsPR1-75 |
| chs-CMA1a | TTACTTAA |  | OsPR1#051, OsPR1-76, OsPR1-71 |
| MYB recognition site | CCGTTG |  | OsPR1#051, OsPR1#101, OsPR1#073, OsPR1-22, OsPR1-73, OsPR1-78, OsPR1-72 |
| MYB | C/TAACCA |  | OsPR1#012, OsPR1#052, OsPR1#022, OsPR1#051, OsPR1#074, OsPR1#121, OsPR1#071, OsPR1#073, OsPR1-21, OsPR1-77, OsPR1-22, OsPR1-61, OsPR1-73, OsPR1-78, OsPR1-72, OsPR1-75, OsPR1-71, OsPR1-74 |
| Myb | TAACTG |  | OsPR1#012, OsPR1#052, OsPR1#021, OsPR1#071, OsPR1-22, OsPR1-73, OsPR1-78, OsPR1-72, OsPR1-75, OsPR1-71, OsPR1-74 |
| WUN-motif | AAATTTCTT |  | OsPR1#072, OsPR1-72 |
| GTGGC-motif | GATTCTGTGGC | light responsive element | OsPR1#074 |
| CCAAT-box | CAACGG | MYBHv1 binding site | OsPR1#051, OsPR1#101, OsPR1#073, |
| LTR | CCGAAA | cis-acting element involved in low-temperature responsiveness | OsPR1#022, OsPR1#073, OsPR1-21, OsPR1-77, OsPR1-22, OsPR1-61, OsPR1-78, OsPR1-75, OsPR1-71 |
| MBS | CAACTG | MYB binding site involved in drought-inducibility | OsPR1#012, OsPR1#052, OsPR1#071, OsPR1-22, OsPR1-73, OsPR1-78, OsPR1-75, OsPR1-71, OsPR1-74 |
| CCAAT-box | CAACGG | MYBHv1 binding site | OsPR1#051, OsPR1#101, OsPR1#073, OsPR1-72 |
| TC-rich repeats | GTTTTCTTAC | cis-acting element involved in defense and stress responsiveness | OsPR1#011, OsPR1#012, OsPR1#022, OsPR1#051, OsPR1#052, OsPR1#073, OsPR1#074, OsPR1#121, OsPR1-72 |
| GC-motif | CCCCCG | enhancer-like element involved in anoxic specific inducibility | OsPR1#011, OsPR1-76, OsPR1-78, OsPR1-72 |
| as-1 | TGACG |  | OsPR1#011, OsPR1#074, OsPR1#121, OsPR1#012, OsPR1#052, OsPR1#022, OsPR1#021, OsPR1-21, OsPR1-76, OsPR1-77, OsPR1-22, OsPR1-73, OsPR1-72, OsPR1-75, OsPR1-71, OsPR1-74 |
| STRE | AGGGG |  | OsPR1#011, OsPR1#012, OsPR1#022, OsPR1#052, OsPR1#072, OsPR1#101, OsPR1#121, OsPR1-21, OsPR1-76, OsPR1-77, OsPR1-61, OsPR1-72, OsPR1-75, OsPR1-74 |
| Sp1 | GGGCGG | light responsive element | OsPR1-76, OsPR1-61 |
| CCGTCC-box | CCGTCC |  | OsPR1-76 |
| CTAG-motif | ACTAGCAGAA |  | OsPR1-61, OsPR1-78, OsPR1-74 |
| AC-I | (T/C)C(T/C)(C/T)ACC(T/C)ACC |  | OsPR1-76 |
| **HORMONE** |  |  |  |
| TGACG-motif | TGACG | MeJA-responsiveness | OsPR1#011, OsPR1#012, OsPR1#021, OsPR1#022, OsPR1#052, OsPR1#074, OsPR1#121, OsPR1-21, OsPR1-76, OsPR1-77, OsPR1-22, OsPR1-73, OsPR1-72, OsPR1-75, OsPR1-71, OsPR1-74 |
| CGTCA-motif | CGTCA | MeJA-responsiveness | OsPR1#011, OsPR1#012, OsPR1#021, OsPR1#022, OsPR1#052, OsPR1#074, OsPR1#121, OsPR1-21, OsPR1-76, OsPR1-77, OsPR1-22, OsPR1-73, OsPR1-72, OsPR1-75, OsPR1-71, OsPR1-74 |
| TCA-element | CCATCTTTTT | cis-acting element involved in salicylic acid responsiveness | OsPR1#011, OsPR1#051, OsPR1#121, OsPR1#072, OsPR1#052, OsPR1#073, OsPR1-77, OsPR1-73, OsPR1-71, OsPR1-74, OsPR1-72 |
| GARE-motif | TCTGTTG | gibberellin-responsive element | OsPR1#051, OsPR1-78, OsPR1-74 |
| P-box | CCTTTTG | gibberellin-responsive element | OsPR1-61, OsPR1-74 |
| ABRE | CGTACGTGCA | cis-acting element involved in the abscisic acid responsiveness | OsPR1#011, OsPR1#012, OsPR1#022, OsPR1#051, OsPR1#052, OsPR1#071, OsPR1#072, OsPR1#074, OsPR1#121, OsPR1-21, OsPR1-77, OsPR1-78, OsPR1-72, OsPR1-75, OsPR1-71 |
| AAGAA-motif | gGTAAAGAAA |  | OsPR1#022, OsPR1#071, OsPR1#073,  OsPR1#101, OsPR1#121, OsPR1-61, OsPR1-73, OsPR1-72, OsPR1-74 |
| AuxRR-core | GGTCCAT | auxin responsiveness | OsPR1#012, OsPR1-21 |
| TGA-element | AACGAC | auxin-responsive element | OsPR1#051, OsPR1#071, OsPR1#072, OsPR1#121, OsPR1-21, OsPR1-76, OsPR1-73, OsPR1-78 |
| ERE | ATTTTAAA |  | OsPR1#071, OsPR1-22, OsPR1-73, OsPR1-72, OsPR1-71 |
| E2Fb | TTTGCCGC |  | OsPR1#012, OsPR1#101 |
| DRE core | GCCGAC |  | OsPR1-76, OsPR1-22, OsPR1-74 |
| DRE1 | ACCGAGA |  | OsPR1-76 |
| **TISSUE**  **SPECIFIC** |  |  |  |
| CAT-box | GCCACT | meristem expression | OsPR1#021, OsPR1#051, OsPR1#101, OsPR1-21, OsPR1-22, OsPR1-61, |
| dOCT | CACGGATC |  | OsPR1#074, OsPR1#101 |
| CCGTCC-box | CCGTCC |  | OsPR1#051, OsPR1#072, OsPR1#052, OsPR1#073, |
| RY-element | CATGCATG | seed-specific regulation | OsPR1#012, OsPR1#051, OsPR1#071, OsPR1#101, OsPR1#121, OsPR1-77, OsPR1-74, OsPR1-71 |
| MBSI | aaaAaaC(G/C)GTTA | MYB binding site involved in flavonoid biosynthetic | OsPR1#051, OsPR1#051, OsPR1-77 |
| O2-site | GATGA(C/T)(A/G)TG(A/G) | zein metabolism regulation | OsPR1#011, OsPR1#051, OsPR1#073, OsPR1-21, OsPR1-71 |
| circadian | CAAAGATATC | element involved in circadian | OsPR1#012 |
| GCN4_motif | TGAGTCA | endosperm expression | OsPR1#022 |

**Supplementary Table 4: Category wise list of cis-elements extracted from 1000 bp upstream region of *PR1* genes of *Z. mays, Brachypodium distachyon*, *Hordeum vulgare*, and *Brassica rapa*, using PlantCare**

| **STRESS** |  |  |  |
| --- | --- | --- | --- |
| **Motifs** | **Consequence** | **Function** | **Accession** |
| GT1-motif | GGTTAAT | light responsive element | ZMPR1-74, ZmPR1-53, ZmPR1-11, BDPR1-21, BDPR1-11, BDPR1-41, BDPR1-33, HvPR1-11, HvPR1-53, HvPR1-72, HvPR1-51, HvPR1-52, Brara.I05604.1, Brara.I01145.1, Brara.H01250.1, Brara.J00054, Brara.H01563, Brara.K00836, Brara.C04098, Brara.A01573, Brara.B01198, Brara.C01183.1 , Brara.C03754 |
| G-box | TACGTG | light responsiveness | ZMPR1-81, ZmPR1-72, ZmPR1-52, ZmPR1-51, ZmPR1-73, ZmPR1-12, ZmPR1-53, ZmPR1-31, ZmPR1-11, BDPR1-34, BDPR1-21, BDPR1-13, BDPR1-12, BDPR1-11, BDPR1-31, BDPR1-32, BDPR1-22, HVPR1-73, HVPR1-11, HVPR1-55, HVPR1-61, HVPR1-53, HVPR1-54, HVPR1-74, HVPR1-72, HvPR1-52, HVPR1-71, Brara.K00640.1, Brara.I05604.1, Brara.G00102.1, Brara.G00101.1, Brara.A00432.1, Brara.K00636, Brara.H01563, Brara.F00326, Brara.A01572, Brara.C03754, Brara.C04099 |
| CAG-motif | GAAAGGCAGAC | part of a light response element | Brara.G00102.1, Brara.I05604.1 |
| ACE | GACACGTATG | light responsiveness | ZMPR1-74, ZmPR1-72, ZmPR1-12, BDPR1-12, BDPR1-31, BDPR1-13, HVPR1-61, HVPR1-54, HvPR1-51, Brara.K00836, Brara.H01250.1 |
| I-box | cGATAAGGCG | light responsive element | ZMPR1-81, HVPR1-73, HVPR1-74, Brara.K00640.1, Brara.K00636, Brara.B01198, Brara.C01183.1, Brara.C04099 |
| SP1 | GGGCGG | light responsive element | ZmPR1-53, ZmPR1-31, BDPR1-32, BDPR1-14, BDPR1-12, BDPR1-31, BDPR1-21, HVPR1-73, HVPR1-54, Brara.J02861.1 |
| MYC | CATTTG |  | ZMPR1-81, ZMPR1-74, ZmPR1-72, ZmPR1-51, ZmPR1-31, ZmPR1-71,  BDPR1-34, BDPR1-13, BDPR1-14, BDPR1-31, BDPR1-41, BDPR1-22, BDPR1-33, Brara.K00640.1, Brara.J02861.1, Brara.I05604.1, Brara.H01250.1, Brara.G00102.1, Brara.G00101.1, Brara.A00433.1, Brara.K00636, Brara.J00054, Brara.H01563, Brara.F00326, Brara.K00836, Brara.C04099, Brara.C03754, Brara.A01572, Brara.A01573, Brara.B01198, Brara.C01183.1, Brara.C03265, HVPR1-73, HVPR1-11, HVPR1-55, HVPR1-53, HVPR1-54, HVPR1-72, HvPR1-51, HvPR1-52, HVPR1-71 |
| CARE | CAACTCCC |  | ZmPR1-31, BDPR1-32 |
| STRE | AGGGG |  | ZMPR1-74, ZMPR1-81, ZmPR1-72, ZmPR1-51, ZmPR1-73, ZmPR1-12, ZmPR1-11,  BDPR1-34, BDPR1-13, BDPR1-14, BDPR1-12, BDPR1-11, BDPR1-41, BDPR1-22, BDPR1-33, HVPR1-53, HVPR1-54, HVPR1-72, HvPR1-51, HvPR1-52, HVPR1-71, Brara.A01572, Brara.A01573, Brara.B01198, Brara.C01183.1 |
| MBS | CAACTG | MYB binding site involved in drought-inducibility | ZMPR1-81, ZmPR1-12, ZmPR1-71, BDPR1-34, BDPR1-21, BDPR1-11, BDPR1-31, BDPR1-32, BDPR1-33, HVPR1-73, HVPR1-11, HVPR1-72, HvPR1-52, HVPR1-71, Brara.K00640.1, Brara.I05604.1, Brara.H01250.1, Brara.C03265, Brara.H01347, Brara.K00636 |
| F-box | CTATTCTCATT |  | ZmPR1-52, ZmPR1-53, Brara.H01347, Brara.H01563, |
| GTGGC-motif | GATTCTGTGGC | part of a light responsive element | ZmPR1-52, BDPR1-14 |
| LTR | CCGAAA | low-temperature responsiveness | ZmPR1-52, ZmPR1-53, BDPR1-34, BDPR1-14, BDPR1-31, BDPR1-33, HVPR1-55, HVPR1-72, HvPR1-52, HVPR1-71, Brara.J00054, Brara.J02861.1, Brara.G00101.1, Brara.A00433.1, Brara.B01198 |
| ARE | AAACCA | anaerobic induction | ZMPR1-81, ZmPR1-72, ZmPR1-51, ZmPR1-73, ZmPR1-12, ZmPR1-53, ZmPR1-31, BDPR1-12, BDPR1-31, BDPR1-21, BDPR1-32, BDPR1-33, BDPR1-34, BDPR1-13, BDPR1-22, HVPR1-73, HVPR1-55, HVPR1-74, HVPR1-53, HvPR1-51, HvPR1-52, Brara.I05604.1, Brara.I01145.1, Brara.H01250.1, Brara.G00102.1, Brara.G00101.1, Brara.A00432.1, Brara.H01347, Brara.K00836, Brara.C04098, Brara.C03754, Brara.A01573, Brara.A00713, Brara.B01198, Brara.C03265, Brara.H01563 |
| ATCT-motif | AATCTAATCC | light responsiveness | ZmPR1-51, BDPR1-12, BDPR1-41, Brara.K00640.1, Brara.I01145.1, Brara.A01572, Brara.H01347, Brara.K00636 |
| ATC-motif | AGTAATCT | light responsiveness | ZmPR1-12, ZmPR1-11, Brara.C04099, Brara.H01347 |
| 3-AF1 binding site | TAAGAGAGGAA | light responsiveness | ZmPR1-73, Brara.J00054 |
| GA-motif | ATAGATAA | light responsiveness | ZmPR1-73, ZmPR1-53, Brara.H01347 |
| Box 4 | ATTAAT | light responsiveness | ZmPR1-51, ZmPR1-73, ZmPR1-12, ZmPR1-53, ZmPR1-31, BDPR1-21, BDPR1-13, BDPR1-14, BDPR1-11, BDPR1-31, BDPR1-32, BDPR1-22, HVPR1-73, HVPR1-11, HVPR1-74, HvPR1-51, HVPR1-72.1, HvPR1-52, Brara.K00640.1, Brara.I05604.1, Brara.H01250.1, Brara.G00102.1, Brara.G00101.1, Brara.A00433.1, Brara.A00432.1, Brara.H01347, Brara.F00326, Brara.C04099, Brara.C04098, Brara.A01573, Brara.A00713, Brara.B01198, Brara.C03754, Brara.K00636, |
| Box II | TGGTAATAA | part of a light responsive element | ZmPR1-51, ZmPR1-53, BDPR1-12, HVPR1-53, Brara.A01573, Brara.A00713 |
| W box | TTGACC |  | ZMPR1-81, ZMPR1-74, ZmPR1-72, ZmPR1-73, ZmPR1-53, BDPR1-12, BDPR1-11, HVPR1-73, HVPR1-55, HVPR1-61, HVPR1-54, HVPR1-71, Brara.I05604.1, Brara.H01347, Brara.F00326, Brara.B01198, Brara.C04099, Brara.K00836 |
| MRE | AACCTAA | MYB binding site involved in light responsiveness | ZmPR1-72, ZmPR1-11, BDPR1-33, HvPR1-52, Brara.J02861.1, Brara.I01145.1, Brara.A01572, Brara.C01183.1, Brara.C03265 |
| MYB-like sequence | TAACCA |  | ZMPR1-81, ZMPR1-74, ZmPR1-72, ZmPR1-73, ZmPR1-12, ZmPR1-53, BDPR1-12, BDPR1-41, BDPR1-34, HVPR1-53, HVPR1-72, HVPR1-73, HvPR1-52, Brara.J02861.1, Brara.I01145.1, Brara.H01250.1, Brara.G00101.1, Brara.J00054, Brara.C03754, Brara.A01572, Brara.A01573, Brara.B01198, Brara.C03265 |
| MYB recognition site | CCGTTG |  | ZMPR1-81, ZmPR1-52, ZmPR1-12, ZmPR1-31, ZmPR1-11, BDPR1-34, BDPR1-21, BDPR1-14, BDPR1-33, HVPR1-61, HVPR1-72, HVPR1-71, Brara.I05604.1, Brara.C03754, Brara.C04098, Brara.A00432.1 |
| Myb-binding site | CAACAG |  | Brara.C04099, Brara.C04098, Brara.I01145.1 |
| MYB | C/TAACCA |  | ZMPR1-81, ZmPR1-72, ZMPR1-74, ZmPR1-52, ZmPR1-51, ZmPR1-73, ZmPR1-12, ZmPR1-53, BDPR1-34, BDPR1-22, BDPR1-21, BDPR1-12, BDPR1-11, BDPR1-32, BDPR1-41, BDPR1-33, HVPR1-53, HVPR1-74, HVPR1-61, HVPR1-72, HVPR1-11, HVPR1-73, HvPR1-52, HVPR1-71, Brara.K00640.1, Brara.J02861.1, Brara.I05604.1, Brara.I01145.1, Brara.H01250.1, Brara.G00101.1, Brara.A00432.1, Brara.K00636, Brara.J00054, Brara.H01563, Brara.F00326, Brara.C04099, Brara.C04098, Brara.C03754, Brara.C03265, Brara.A01572, Brara.A01573, Brara.A00713, Brara.B01198, Brara.C01183.1 |
| Myb | TAACTG |  | ZMPR1-81, ZmPR1-52, ZmPR1-73, ZmPR1-12, ZmPR1-71, BDPR1-34, BDPR1-13, BDPR1-22, BDPR1-41, BDPR1-33, BDPR1-12, BDPR1-11, BDPR1-31, BDPR1-21, HVPR1-73, HVPR1-11, HVPR1-54, HVPR1-61, HVPR1-72, HvPR1-52, HVPR1-71, Brara.J02861.1, Brara.I05604.1, Brara.I01145.1, Brara.H01250.1, Brara.G00101.1, Brara.A00433.1, Brara.A00432.1, Brara.H01347, Brara.C03754, Brara.A00713, Brara.B01198, Brara.C03265 |
| AT-rich sequence | TAAAATACT | element for maximal elicitor-mediated activation | Brara.B01198, Brara.C04098, Brara.F00326, Brara.H01563, |
| WUN-motif | AAATTTCTT |  | BDPR1-12, Brara.K00640.1, Brara.H01250.1, Brara.G00102.1, Brara.K00636, Brara.J00054, Brara.H01563, Brara.H01347, Brara.A01573, Brara.B01198, Brara.C04098, Brara.H01347 |
| CCAAT-box | CAACGG | MYBHv1 binding site | ZMPR1-81, ZmPR1-12, ZmPR1-11, BDPR1-34, BDPR1-21, BDPR1-14, BDPR1-33, Brara.I05604.1, HVPR1-61, HVPR1-72, Brara.C03754, Brara.A00432.1, Brara.I05604.1 |
| CTAG-motif | ACTAGCAGAA |  | ZmPR1-72 |
| as-1 | TGACG |  | ZMPR1-81, ZmPR1-51, ZmPR1-71, ZmPR1-72, ZMPR1-74, ZmPR1-52, ZmPR1-73, ZmPR1-53, ZmPR1-11, BDPR1-13, BDPR1-12, BDPR1-31, BDPR1-21, BDPR1-34, HVPR1-55, HVPR1-61, HVPR1-54, HVPR1-74, HvPR1-52, HVPR1-71 |
| GC-motif | CCCCCG | anoxic specific inducibility | ZMPR1-74, ZmPR1-53, BDPR1-33, HVPR1-61, HVPR1-53, HVPR1-54, HvPR1-51, Brara.G00102.1 |
| LAMP-element | CTTTATCA | light responsive element | ZmPR1-72, Brara.J02861.1 |
| WRE3 | CCACCT |  | ZmPR1-31, ZmPR1-11, ZmPR1-71, BDPR1-12, BDPR1-32, HVPR1-74, HVPR1-61, HvPR1-51, HvPR1-52, Brara.I01145.1, Brara.H01250.1, Brara.G00101.1, Brara.C01183.1, Brara.C03265 |
| AC-I | (T/C)C(T/C)(C/T)ACC(T/C)ACC |  | BDPR1-31, BDPR1-22 |
| TCCC-motif | TCTCCCT | light responsive element | BDPR1-41, HVPR1-74, Brara.C01183.1 |
| TCT-motif | TCTTAC | light responsive element | BDPR1-21, BDPR1-41, BDPR1-22, HVPR1-61, HVPR1-74, HVPR1-71, Brara.K00640.1, Brara.A00432.1, Brara.K00636, Brara.C03265, Brara.C04098, Brara.C04099 |
| GATA-motif | GATAGGG | light responsive element | BDPR1-13, BDPR1-12, BDPR1-11, HVPR1-72, HvPR1-52, HVPR1-71, Brara.I01145.1, Brara.J00054, Brara.H01563, Brara.A01572, Brara.B01198, Brara.C01183.1, Brara.C04099 |
| AE-box | AGAAACAA | light response | BDPR1-31, HVPR1-73, HVPR1-71, Brara.I01145.1, Brara.J00054, Brara.A01572, Brara.C04098, Brara.F00326, Brara.H01563 |
| TC-rich repeats | GTTTTCTTAC | defense and stress responsiveness | BDPR1-34, BDPR1-14, HVPR1-11, HVPR1-55, HVPR1-53, HVPR1-74, HvPR1-51, , HvPR1-52, Brara.J02861.1, Brara.A00432.1, Brara.C03265, Brara.C04098 |
| chs-CMA1a | TTACTTAA | light responsive element | HvPR1-52, Brara.I05604.1, Brara.H01347, Brara.G00102.1, Brara.I01145.1 |
| **HORMONE** |  |  |  |
| TGACG-motif | TGACG | MeJA-responsiveness | ZMPR1-81, ZMPR1-74, ZmPR1-72, ZmPR1-52, ZmPR1-51, ZmPR1-73, ZmPR1-53, ZmPR1-11, ZmPR1-71, BDPR1-34, BDPR1-21, BDPR1-13, BDPR1-14, BDPR1-12, BDPR1-31, BDPR1-32, HVPR1-73, HVPR1-11, HVPR1-55, HVPR1-61, HVPR1-74, HVPR1-72, HvPR1-51, HvPR1-52, HVPR1-54.3, HVPR1-71, Brara.K00640.1, Brara.H01250.1, Brara.G00102.1, Brara.G00101.1, Brara.A00433.1, Brara.A00432.1, Brara.K00636, Brara.J00054, Brara.A01572, Brara.A00713, Brara.C01183.1, Brara.H01563 |
| CGTCA-motif | CGTCA | MeJA-responsiveness | ZMPR1-81, ZMPR1-74, ZmPR1-51, ZmPR1-73, ZmPR1-53, ZmPR1-11, ZmPR1-71, BDPR1-34, BDPR1-21, BDPR1-13, BDPR1-14, BDPR1-31, BDPR1-32, HVPR1-73, HVPR1-11, HVPR1-55, HVPR1-61, HVPR1-54, HVPR1-74, HVPR1-72, HvPR1-51, HvPR1-52, HVPR1-71, Brara.K00640.1, Brara.I05604.1, Brara.H01250.1, Brara.G00102.1, Brara.G00101.1, Brara.A00433.1, Brara.K00636, Brara.J00054, Brara.A01572, Brara.A00713, Brara.C01183.1, Brara.H01563 |
| ABRE | CGTACGTGCA | abscisic acid responsiveness | ZMPR1-81, ZmPR1-52, ZmPR1-51, ZmPR1-73, ZmPR1-31, ZmPR1-71, BDPR1-34, BDPR1-21, BDPR1-13, BDPR1-12, BDPR1-11, BDPR1-31, BDPR1-32, HVPR1-73, HVPR1-61, HVPR1-53, HVPR1-54, HVPR1-74, HVPR1-72, HvPR1-51, HvPR1-52, HVPR1-71, Brara.I05604.1, Brara.G00101.1, Brara.A00432.1, Brara.H01563, Brara.A01573, Brara.C03754, Brara.C04099, Brara.F00326, Brara.G00102.1 |
| ABRE3a | TACGTG |  | Brara.G00101.1, Brara.G00102.1 |
| ABRE4 | CACGTA |  | Brara.G00101.1, Brara.G00102.1 |
| TCA-element | CCATCTTTTT | salicylic acid responsiveness | ZMPR1-81, ZmPR1-72, ZmPR1-11, ZmPR1-71, BDPR1-12, BDPR1-31, BDPR1-13, HvPR1-51, Brara.A00433.1, Brara.A00432.1, Brara.B01198, Brara.C03265, Brara.K00836 |
| TGA-element | AACGAC | auxin-responsive element | ZmPR1-12, ZmPR1-11, BDPR1-34, BDPR1-21, BDPR1-31, BDPR1-41, HVPR1-73, HVPR1-55, Brara.K00640.1, Brara.I01145.1, Brara.K00636, Brara.J00054, Brara.A00713, Brara.H01347, Brara.H01563 |
| P-box | CCTTTTG | gibberellin-responsive element | ZMPR1-74, ZmPR1-73, ZmPR1-53, BDPR1-12, BDPR1-33, HVPR1-11, Brara.G00101.1, Brara.H01347, Brara.C04098, Brara.C04099, Brara.K00836 |
| DRE1 | ACCGAGA |  | ZmPR1-72, ZmPR1-73, BDPR1-31, HVPR1-73, HVPR1-72, HvPR1-51, HvPR1-52 |
| DRE core | GCCGAC |  | ZmPR1-11, BDPR1-32, HVPR1-61, HVPR1-54, HVPR1-72, Brara.J02861.1, Brara.A00432.1, Brara.H01250.1 |
| AuxRR-core | GGTCCAT | auxin responsiveness | ZmPR1-72, BDPR1-41, BDPR1-31, BDPR1-34, |
| GARE Motif |  | gibberellin-responsive element | ZmPR1-52, BDPR1-32, BDPR1-33, BDPR1-11, BDPR1-21, BDPR1-22, Brara.C04099, Brara.I01145.1 |
| TATC-box | TATCCCA | gibberellin-responsiveness | BDPR1-41, HVPR1-72, HVPR1-74, Brara.B01198 |
| AAGAA-motif | gGTAAAGAAA |  | BDPR1-34, BDPR1-12, BDPR1-11, BDPR1-32, BDPR1-41, BDPR1-22, Brara.J02861.1, Brara.I05604.1, Brara.A00433.1, Brara.H01563, Brara.H01347, Brara.C04099, Brara.B01198, Brara.C01183.1, Brara.C03265, Brara.C04098 |
| ERE | ATTTCATA | Ethylene responsive element | ZmPR1-51, ZmPR1-12, ZmPR1-53, Brara.K00640.1, Brara.J02861.1, Brara.G00101.1, Brara.A00433.1, Brara.A00432.1, Brara.K00636, Brara.H01347, Brara.C04099, Brara.A01572, Brara.A01573, Brara.B01198, Brara.C03754 |
| TGA | ATTTCATA |  | ZmPR1-73 |
| **TISSUE**  **SPECIFIC** |  |  |  |
| CAT-box | GCCACT | meristem expression | ZMPR1-74, ZmPR1-52, ZmPR1-73, ZmPR1-31, ZmPR1-71, BDPR1-22, BDPR1-33, HVPR1-73, HVPR1-11, HVPR1-53, HVPR1-74, HVPR1-72, HvPR1-52, HVPR1-71, Brara.A01573, Brara.B01198, Brara.C03754, Brara.C04098 |
| RY-element | CATGCATG | seed-specific regulation | ZmPR1-12, ZmPR1-31, BDPR1-12, BDPR1-11, BDPR1-33, HVPR1-11, HVPR1-53, HVPR1-72, HVPR1-71 |
| O2-site | GATGA(C/T)(A/G)TG(A/G) | zein metabolism regulation | ZMPR1-81, ZmPR1-72, ZmPR1-52, BDPR1-34, BDPR1-32, HVPR1-11, HVPR1-61, HVPR1-54, Brara.I05604.1, Brara.G00102.1, Brara.H01563, Brara.A00432.1 |
| MSA-like | T/C)C(T/C)AACGG(T/C)(T/C)A | cell cycle regulation | ZmPR1-52, BDPR1-14, |
| MBSI | aaaAaaC(G/C)GTTA | MYB binding site involved in flavonoid biosynthetic genes regulation | Brara.K00640.1 |
| AP-1 | TGAGTTAG | Cell proliferation | Brara.J02861.1 |
| CCGTCC-box | CCGTCC |  | ZmPR1-73, BDPR1-12, BDPR1-11, BDPR1-33 |
| circadian | CAAAGATATC | circadian | BDPR1-33, HVPR1-53, Brara.F00326, Brara.I05604.1 |
| GCN4_motif | TGAGTCA | endosperm expression | BDPR1-33, HVPR1-73, HVPR1-55, HVPR1-54, Brara.J00054 |
| NON | CAACGGCCACG |  | BDPR1-34, BDPR1-33, |
| dOCT | CACGGATC |  | HVPR1-61 |

**Supplementary Table 5: List of Genes taken for Search BLASTP search of other genes**

| **Protein ID** | **MSU (LOC_OsID)** |
| --- | --- |
| LOC_OS01G28450.1 (OsPR1#011) | LOC_Os01g28450.1 |
| LOC_OS01G28500.1 (OsPR1# 012) | LOC_Os01g28500.1 |
| LOC_OS02G54540.1 (OsPR1# 021) | LOC_Os02g54540.1 |
| LOC_OS02G54560.1 (OsPR1# 022) | LOC_Os02g54560.1 |
| LOC_OS05G51660.1 (OsPR1# 051) | LOC_Os05g51660.1 |
| LOC_OS05G51680.1 (OsPR1# 052) | LOC_Os05g51680.1 |
| LOC_OS07G03279.1 (OsPR1# 071) | LOC_Os07g03279.1 |
| LOC_OS07G03580.1 (OsPR1# 072) | LOC_Os07g03580.1 |
| LOC_OS07G03590.1 (OsPR1# 073) | LOC_Os07g03590.1 |
| LOC_OS07G03710.1 (OsPR1# 074) | LOC_Os07g03710.1 |
| LOC_OS10G11500.1 (OsPR1# 101) | LOC_Os10g11500.1 |
| LOC_OS12G43700.1 (OsPR1# 121) | LOC_Os12g43700.1 |

**Supplementary Table 6: List of genes obtained after BLASTP search**

| ***O.sativa*** | ***H.Vulgare*** | ***Z.mays*** | ***B.rapa*** | ***B. distachyon*** |
| --- | --- | --- | --- | --- |
| LOC_Os01g28450.1 | HV_Q05968 | ZM_ACJ62559 | BR_VCD84050 | BD_XP_003557605 |
| LOC_Os01g28500.1 | HV_KAE8807165 | ZM_XP_008651988 | BR_XP_009135645 | BD_KQK20924 |
| LOC_Os02g54540.1 | HV_BAK01044 | ZM_ONM08903.1 | BR_XP_009136247 | BD_XP_003561249 |
| LOC_Os02g54560.1 | HV_KAE8813015 | ZM_ONL98986.1 | BR_AAT46023 | BD_XP_003572883 |
| LOC_Os05g51660.1 | HV_KAE8806578 | ZM_AQK75611 | BR_XP_009136246 | BD_KQK20923 |
| LOC_Os05g51680.1 | HV_KAE8766408 | ZM_AQK75612 | BR_VDC65007 | BD_XP_003561435 |
| LOC_Os07g03279.1 | HV_KAE8810549 | ZM_AQK75613 | BR_XP_009137911 | BD_KQK02081 |
| LOC_Os07g03580.1 | HV_KAE8805026 | ZM_ONM51204 | BR_XP_009127912 | BD_XP_003573142 |
| LOC_Os07g03590.1 |  | ZM_ONM51205 | BR_XP_009109192 | BD_XP_014756083 |
| LOC_Os07g03710.1 |  | ZM_ONM52792 | BR_RID78779 | BD_XP_003579097 |
| LOC_Os10g11500.1 |  | ZM_ONM32708 | BR_VDD16626 | BD_KQK04554 |
| LOC_Os12g43700.1 |  |  | BR_VDC81544 | BD_XP_003572884 |
| LOC_Os02g54530.1 |  |  | BR_VDC83896 | BD_PNT69320 |
| LOC_Os02g54570.1 |  |  | BR_RID78780 | BD_XP_010230947 |
| LOC_Os06g24290.1 |  |  | BR_RID77527 |  |
| LOC_Os07g03409.1 |  |  | BR_RID77528 |  |
| LOC_Os07g03600.1 |  |  | BR_VDC86616 |  |
| LOC_Os07g03680.1 |  |  | BR_RID69062 |  |
| LOC_Os07g03690.1 |  |  | BR_RIA05031 |  |
| LOC_Os07g03730.1 |  |  | BR_RIA05035 |  |
| LOC_Os07g03740.1 |  |  | BR_RID52650 |  |
| LOC_Os07g14030.1 |  |  | BR_RID52651 |  |
| LOC_Os07g14070.1 |  |  | BR_RID50524 |  |
|  |  |  | BR_RID50861 |  |
|  |  |  | BR_RID44342 |  |
|  |  |  | BR_RID43023 |  |

**Supplementary Table 7: List of Genes obtained after HMM search**

| ***O.sativa*** | ***H.Vulgare*** | ***Z.mays*** | ***B.rapa*** |
| --- | --- | --- | --- |
| LOC_Os12g43700.1 | HORVU5Hr1G001690.1 | Zm00001d018738_P001 | Brara.C01183.1.p |
| LOC_Os05g51680.2 | HORVU5Hr1G001690.2 | Zm00001d018734_P001 | Brara.B01198.1.p |
| LOC_Os05g51680.1 | HORVU1Hr1G095440.1 | Zm00001d029558_P001 | Brara.A01573.1.p |
| LOC_Os02g27310.1 | HORVU7Hr1G040740.4 | Zm00001d009296_P001 | Brara.H01563.1.p |
| LOC_Os02g54560.1 | HORVU5Hr1G055950.2 | Zm00001d018322_P001 | Brara.K00640.1.p |
| LOC_Os10g11500.1 | HORVU5Hr1G055950.1 | Zm00001d019364_P001 | Brara.A01572.1.p |
| LOC_Os01g28500.1 | HORVU5Hr1G056040.1 | Zm00001d018323_P001 | Brara.J00054.1.p |
| LOC_Os01g28450.1 | HORVU7Hr1G040730.1 | Zm00001d041230_P001 | Brara.I05604.1.p |
| LOC_Os07g03710.1 | HORVU7Hr1G040730.2 | Zm00001d018737_P001 | Brara.A00713.1.p |
| LOC_Os07g03730.1 | HORVU7Hr1G033530.3 | Zm00001d018321_P001 | Brara.K00836.1.p |
| LOC_Os07g03279.1 | HORVU7Hr1G033530.4 | Zm00001d004089_P001 | Brara.A00433.1.p |
| LOC_Os07g03458.1 | HORVU5Hr1G001720.2 | Zm00001d039212_P001 | Brara.C03754.1.p |
| LOC_Os07g03368.1 | HORVU7Hr1G033530.1 | Zm00001d009772_P001 | Brara.C04099.1.p |
| LOC_Os07g03467.1 | HORVU7Hr1G033620.1 | Zm00001d018324_P001 | Brara.C03265.1.p |
| LOC_Os07g03590.1 | HORVU5Hr1G106010.1 | Zm00001d052068_P001 | Brara.C04098.1.p |
| LOC_Os07g03288.1 | HORVU5Hr1G106010.3 | Zm00001d033902_P001 | Brara.F00327.1.p |
| LOC_Os07g03377.1 | HORVU5Hr1G106010.2 |  | Brara.K01652.1.p |
| LOC_Os07g03690.1 | HORVU5Hr1G106020.1 | ***B. distachyon*** | Brara.J02861.1.p |
| LOC_Os04g22330.1 | HORVU7Hr1G022230.1 | Bradi3g60230.1.p | Brara.K00639.1.p |
| LOC_Os04g22340.1 | HORVU5Hr1G001720.1 | Bradi3g53630.1.p | Brara.F00326.1.p |
| LOC_Os03g52300.1 | HORVU7Hr1G040740.1 | Bradi1g57580.1.p | Brara.K00636.1.p |
| LOC_Os02g54540.1 | HORVU2Hr1G092060.1 | Bradi2g14240.1.p | Brara.H01347.1.p |
| LOC_Os05g51660.1 | HORVU3Hr1G051110.2 | Bradi1g57540.1.p | Brara.H01250.1.p |
| LOC_Os07g03580.1 | HORVU3Hr1G051110.3 | Bradi1g57590.1.p | Brara.A00432.1.p |
| LOC_Os07g03319.1 | HORVU3Hr1G051110.1 | Bradi4g00865.1.p | Brara.I01145.1.p |
| LOC_Os07g03409.1 | HORVU3Hr1G114820.1 | Bradi1g12360.1.p | Brara.G00102.1.p |
| LOC_Os07g03499.1 | HORVU0Hr1G007430.1 | Bradi3g60260.2.p | Brara.G00101.1.p |
| LOC_Os07g03740.1 | HORVU0Hr1G026540.1 | Bradi3g53637.1.p | Brara.K00635.1.p |
| LOC_Os02g54530.1 | HORVU3Hr1G114790.1 | Bradi4g38910.1.p | Brara.J01172.1.p |
| LOC_Os02g27300.1 | HORVU0Hr1G026530.1 | Bradi2g14256.1.p |  |
| LOC_Os07g03750.1 | HORVU0Hr1G026520.1 | Bradi1g09637.1.p |  |
| LOC_Os02g54570.1 | HORVU6Hr1G083470.1 | Bradi3g53681.1.p |  |
| LOC_Os04g22210.1 | HORVU6Hr1G083310.2 | Bradi1g57575.1.p |  |
| LOC_Os07g03680.1 | HORVU6Hr1G083310.1 |  |  |
| LOC_Os07g14070.1 | HORVU7Hr1G040740.5 |  |  |
| LOC_Os07g03620.1 | HORVU4Hr1G010540.1 |  |  |
| LOC_Os07g03610.1 | HORVU6Hr1G083350.1 |  |  |
| LOC_Os07g14030.1 | HORVU7Hr1G031250.1 |  |  |
| LOC_Os04g22230.1 | HORVU7Hr1G040740.3 |  |  |
| LOC_Os04g22220.1 | HORVU2Hr1G014500.1 |  |  |
|  | HORVU0Hr1G035110.1 |  |  |
|  | HORVU7Hr1G040730.1 |  |  |
|  | HORVU7Hr1G040730.2 |  |  |
|  | HORVU3Hr1G115900.1 |  |  |
